# Endothelial GDF15 deficiency enhances barrier function and mitigates pulmonary fibrosis

**DOI:** 10.1172/jci.insight.200198

**Published:** 2026-04-23

**Authors:** Kristen Raffensperger, Marta Bueno, Brian J. Philips, Megan Miller, Máté Katona, Shuai Yuan, Adriana Estrada-Bernal, Byron Chuan, Pavan Suresh, Stephanie Taiclet, Scott Hahn, Yingze Zhang, Jonathan K. Alder, Seyed Mehdi Nouraie, Daniel J. Kass, Oliver Eickelberg, Adam C. Straub

**Affiliations:** 1 Pulmonary and Critical Care Medicine, UPMC, Pittsburgh, Pennsylvania, USA.; 2 Division of Pulmonary, Allergy, Critical Care, and Sleep Medicine, Department of Medicine,; 3 Heart, Lung, Blood and Vascular Medicine Institute, and; 4 Department of Pharmacology and Chemical Biology, University of Pittsburgh, Pittsburgh, Pennsylvania, USA.

**Keywords:** Cell biology, Pulmonology, Vascular biology, Calcium signaling, Endothelial cells, Fibrosis

## Abstract

Pulmonary fibrosis is frequently accompanied by pulmonary hypertension, which can occur disproportionate to the extent of fibrosis, suggesting a fibrosis-independent vascular remodeling process. Here, we demonstrated that plasma growth differentiation factor 15 (GDF15) is elevated across diverse fibrotic lung disease subtypes and correlates with markers of elevated right heart pressures but not pulmonary function indices, indicating a possible link to endothelial cell dysfunction. To investigate the import of endothelial GDF15 as a modifier of lung fibrosis pathogenesis, we generated endothelial cell–specific *Gdf15*-KO mice, which showed protection from bleomycin-induced lung injury and fibrosis, with preserved lung function. RNA-seq of human pulmonary microvascular endothelial cells revealed altered expression of barrier-regulatory genes in *GDF15*-deficient endothelial cells compared with controls. Functional studies confirmed that *GDF15* knockdown attenuates thrombin-induced barrier disruption by reducing cytosolic Ca^2+^ responses. Together, these findings implicate endothelial GDF15 as a modifier of vascular permeability and Ca^2+^ signaling and a contributor to lung injury and fibrosis.

## Introduction

Interstitial lung disease (ILD) comprises myriad disorders marked by interstitial inflammation and fibrosis. These include autoimmune conditions, hypersensitivity pneumonitis, and sarcoidosis, as well as idiopathic pulmonary fibrosis (IPF), diagnosed when no clear etiology is identified (1). Pulmonary vasculature involvement in ILD ranges from minimal in IPF to predominant in scleroderma (2), where pulmonary hypertension (PH) may occur in the absence of substantial fibrosis (3, 4).

In IPF, ~30% of patients develop PH (5). Traditionally, this is attributed to structural changes in the parenchyma, with fibrotic remodeling compressing the pulmonary microvasculature with resultant increased right-heart pressures. However, a subset of patients with IPF presents with PH that is disproportionately severe relative to fibrotic burden (6). Similar patterns are observed in chronic obstructive pulmonary disease (COPD), where some individuals develop severe PH despite relatively mild parenchymal disease (7). Importantly, the development of PH in these settings is strongly associated with worsened clinical outcomes (8, 9), prompting investigations into whether a primary vasculopathic process contributes to disease pathogenesis in ILD.

Growth differentiation factor 15 (GDF15), also known as macrophage inhibitory cytokine 1, is elevated across various ILD subtypes, such as IPF (10) and scleroderma (11, 12). GDF15 is a member of the TGFβ superfamily, characterized by a classical cysteine knot (13); it is synthesized as a prepropeptide comprising a signal sequence, a 167–amino acid prodomain, and a 112–amino acid mature domain (14), and forms disulfide-linked homodimers in both its pro- and mature forms (15). Pro-GDF15 can translocate to the nucleus where it inhibits SMAD-mediated transcription or is secreted extracellularly by Exportin 1 (16). Once secreted, pro-GDF15 anchors to the extracellular matrix (17) and is proteolytically cleaved by furin-like enzymes to release the active 24-kDa mature dimer (18).

Basal *GDF15* expression is low to absent in most healthy adult human tissue, with the placenta being a notable exception (15). Its only currently known receptor, GDNF family receptor α-like (GFRAL), is predominantly expressed in the hindbrain and minimally present in peripheral tissues in humans (19). Zhang et al. demonstrated increased circulating GDF15 in response to bleomycin challenge in mice (10) and antibody-mediated neutralization attenuates bleomycin-induced collagen deposition (20), implicating GDF15 in pulmonary fibrotic processes. Moreover, GDF15 is upregulated across a wide range of disease aside from pulmonary fibrosis, including diabetes (21), cancer cachexia (22), COPD (23), heart failure (24), and PH (25). Given its broad disease association and, specifically, its upregulation across a spectrum of ILD, we hypothesized that GDF15 is involved at a ubiquitous structure such as the vasculature, contributing to disease progression via pulmonary vascular dysfunction, possibly through a direct impact on pulmonary endothelial function.

## Results

### GDF15 is elevated across a spectrum of pulmonary disease and correlates with surrogate markers of elevated right-sided intracardiac pressures.

**Figure 1:**
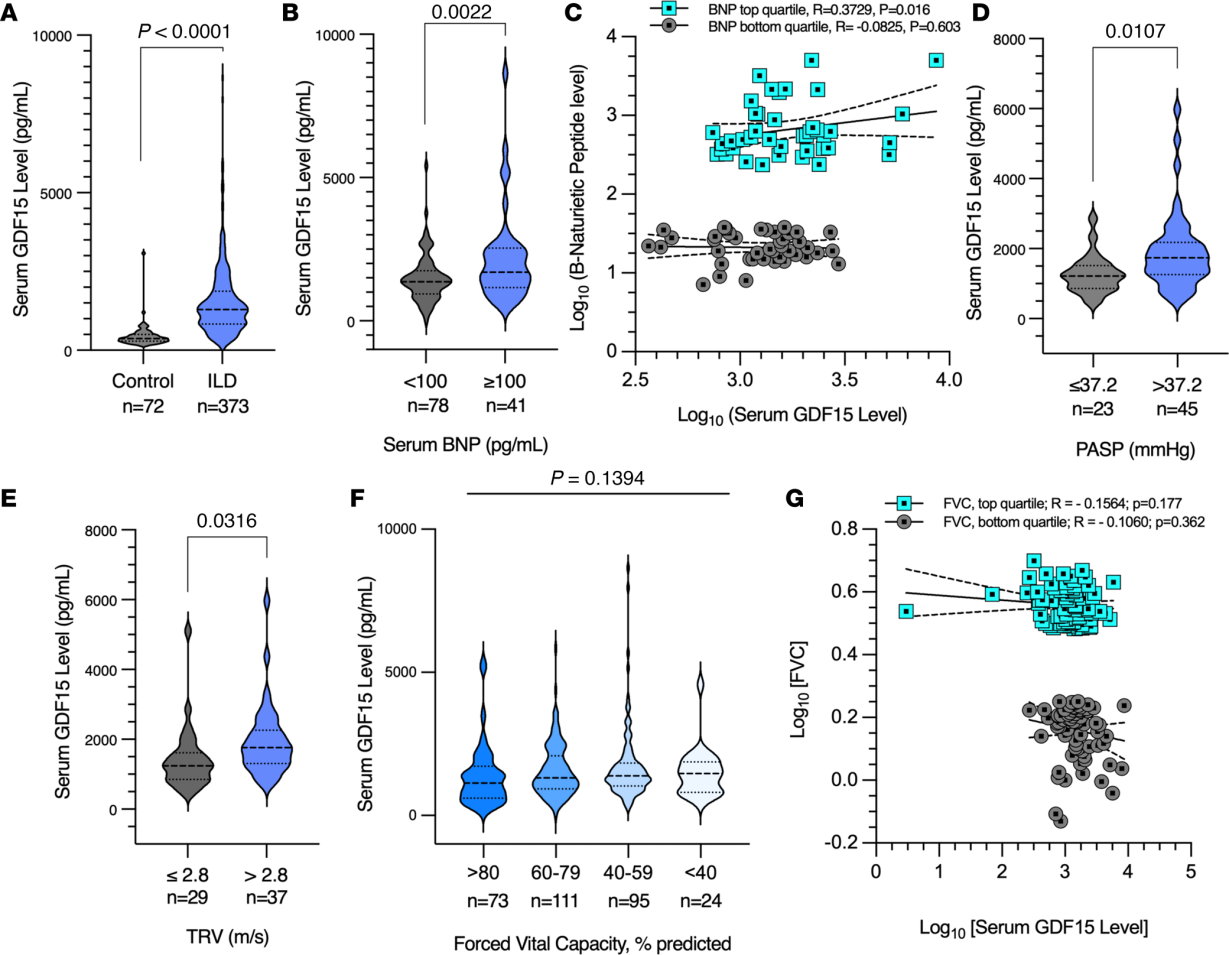
Plasma GDF15 levels positively correlate with markers of right-sided cardiac strain. (**A**) Plasma GDF15 levels in patients with interstitial lung disease (ILD) versus control. (**B**) Plasma GDF15 levels in patients with ILD, separated into 2 groups according to serum B-natriuretic peptide (BNP), using a standard cut-off of 100 ng/mL. Serum BNP levels were drawn within 24 months of GDF15 levels. (**C**) The top and bottom quartiles of serum BNP levels in patients with ILD in correlation with GDF15 levels, displayed with line of best fit and 95% confidence interval. (**D**) Plasma GDF15 levels in patients with ILD separated into 2 groups according to estimated pulmonary arterial systolic pressure (ePASP) on transthoracic echocardiogram (TTE), using a cut-off of 37.2 mmHg. ePASP measurements were acquired within 24 months of GDF15 levels. (**E**) Plasma GDF15 levels in patients with ILD separated into 2 groups according to tricuspid regurgitant velocity (TRV) on TTE, using a cut-off of 2.8 m/s. TRV measurements were acquired within 24 months of GDF15 levels. (**F**) Plasma GDF15 levels in patients with ILD separated into 4 groups according to forced vital capacity (FVC). Pulmonary function data were acquired within 12 months of GDF15 level; if multiple pulmonary function data were available within 12 months of GDF15 level, the set of data acquired closest in time to the date of the GDF15 level was utilized. (**G**) The top and bottom quartile of FVC measurements in patients with ILD plotted against corresponding GDF15 levels, displayed with line of best fit and 95% CI. Violin plots display median and interquartile range. Statistical analyses: unpaired *t* test (**A**, **B**, **D**, and **E**), Spearman correlation (**C** and **G**), and Brown-Forsythe test (**F**).

To query associations of GDF15 in ILD, we utilized a patient cohort comprising multiple subtypes of ILD who were recruited through the Simmons Center for Interstitial Lung Disease in Pittsburgh, Pennsylvania, USA, between January 2003 and October 2016. Of note, prior work by Zhang et al. describes GDF15 levels in the control and IPF subsets of this cohort (10), underscoring alveolar GDF15 secretion under telomere dysfunction and bleomycin challenge. Patient diagnoses in our current study included, but were not limited to, IPF, hypersensitivity pneumonitis, Sjogren’s disease, and scleroderma. Plasma and pulmonary function testing (PFT) data were collected on all patients; a subset of patients underwent additional echocardiographic imaging. Control patients consisted of unrelated healthy patients, randomly recruited, and had no self-reported lung disease. Cohort characteristics are found in Supplemental Table 1 (supplemental material available online with this article; https://doi.org/10.1172/jci.insight.200198DS1). Circulating GDF15 levels in the control cohort correlate with age (Supplemental Figure 1A), recapitulating a relationship already established in prior literature (26). Plasma samples revealed significantly elevated GDF15 levels among patients with ILD (1,522 ± 58 pg/mL) compared with control patients (446 ± 43 pg/mL) (Figure 1A). The ILD cohort was separated by subtype; GDF15 levels remained significantly increased relative to controls for all subtypes except in the case of sarcoidosis (Supplemental Figure 1B).

Next, we examined the relationship between GDF15 and surrogate markers of elevated right heart pressures as well as PFT parameters. The ILD cohort was separated into 2 groups based on standard clinical cut-offs for serum B-natriuretic peptide (BNP), as well as transthoracic echocardiogram (TTE) measurements of estimated pulmonary arterial systolic pressure (ePASP) and tricuspid regurgitant velocity (TRV). Elevated serum BNP was significantly associated with elevated GDF15 (Figure 1B). Additionally, a significant positive correlation existed between BNP and GDF15 among those in the top quartile BNP group (Figure 1C) that was notably absent in the bottom quartile group. Elevated ePASP and TRV also significantly associated with elevated GDF15 levels (Figure 1, D and E). No significant association was identified between GDF15 and forced vital capacity (FVC) (Figure 1, F and G). Furthermore, we found that increased ePASP, but not decreased FVC, on follow-up testing was significantly associated with elevated GDF15 levels (Supplemental Figure 1, C and D). All parameters were reexamined in the IPF subcohort, producing data comparable with those in the pooled ILD cohort (Supplemental Figure 1E). These findings support a potential vascular role for GDF15 in ILD.

### Microvascular changes correlate with GDF15 levels in ILD.

**Figure 2:**
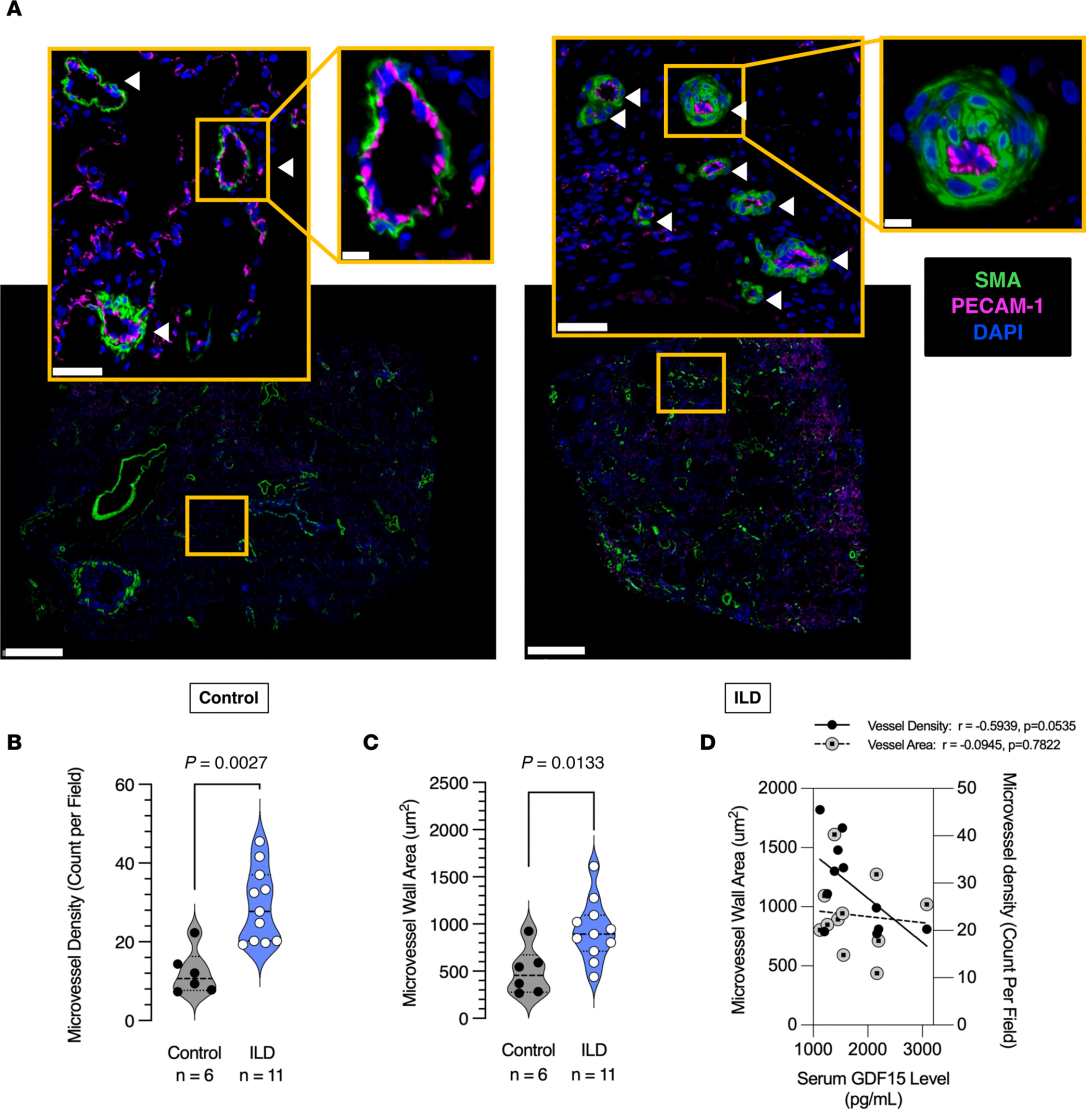
Plasma GDF15 levels correlate with evidence of microvascular change in patients with ILD. (**A**) Representative immunofluorescence images from control and patients with ILD, stained for smooth muscle actin (SMA) shown in green, platelet endothelial cell adhesion molecule-1 (PECAM-1) shown in pink, and DAPI shown in blue. Scale bars: 2 mm (whole lung), 50 μm (partial zoom), and 10 μm (full zoom). White arrows indicate microvessels of interest. Individual vessels are noted with arrowheads. (**B**) Count of vessels with diameter < 50 μm per field, in control and ILD histologic specimens; an average per specimen was calculated from 4 separate fields. (**C**) Vessel wall area of vessels < 50 μm in diameter, in control and ILD histologic specimens. Three vessels were examined per specimen, the average of which was reported as the final value per specimen. (**D**) Plasma GDF15 levels in 11 histologic patients with ILD plotted against each patient’s average microvessel wall area (CI = –0.6571, 0.5357) and average microvessel density (CI = –0.8805, 0.007738). Violin plots display median and interquartile range. Kolmogorov-Smirnov test (**B** and **C**) and Pearson correlation (**D**).

The association of GDF15 with BNP and ePASP, but not with PFT parameters, prompted histologic examination of the pulmonary microvasculature. Histologic lung sections from patients with ILD (*n* = 11) and controls (*n* = 6) were labeled with antibodies against platelet endothelial cell adhesion molecule-1 (PECAM-1) and smooth muscle actin (SMA), and counterstained with DAPI. Representative images from control versus ILD are shown in Figure 2A. In ILD histologic sections, there was increased microvascular density (Figure 2, A and B) as well as increased arteriole wall area (Figure 2, A and C; wall area calculation as per Supplemental Figure 2A), relative to control specimens. A strong negative correlation was found between plasma GDF15 levels and microvessel density in the patients with ILD; no relationship was identified between GDF15 and vessel wall area (Figure 2D). Plasma GDF15 levels of patients with ILD whose histologic sections were examined are displayed in Supplemental Figure 2B, all of which were significantly elevated relative to controls; these data are presented in Figure 1A within the entire ILD cohort. Correlations between GDF15 and BNP, ePASP, and TRV values across the entire cohort can be found in Supplemental Figure 2C, with histologic patient values highlighted. The specific demographics of patients in the histologic cohort can be found in Supplemental Table 2. There was a strong correlation identified between BNP and GDF15 in histologic patients and a moderate correlation between GDF15 and both ePASP and TRV over the entire ILD cohort for those patients whose GDF15 levels were drawn within 24 months of their TTE measurements.

### GDF15 colocalizes with endothelial markers.

To test whether GDF15 would colocalize with the endothelium, histologic sections were stained with antibodies to GDF15, PECAM1, SMA, and DAPI. Representative images from both control and ILD sections are displayed in Supplemental Figure 2D. In both control and patients with ILD, GDF15 colocalizes with endothelial markers.

### Endothelial-specific deletion of GDF15 in mice confers protection against bleomycin-induced lung fibrosis.

**Figure 3:**
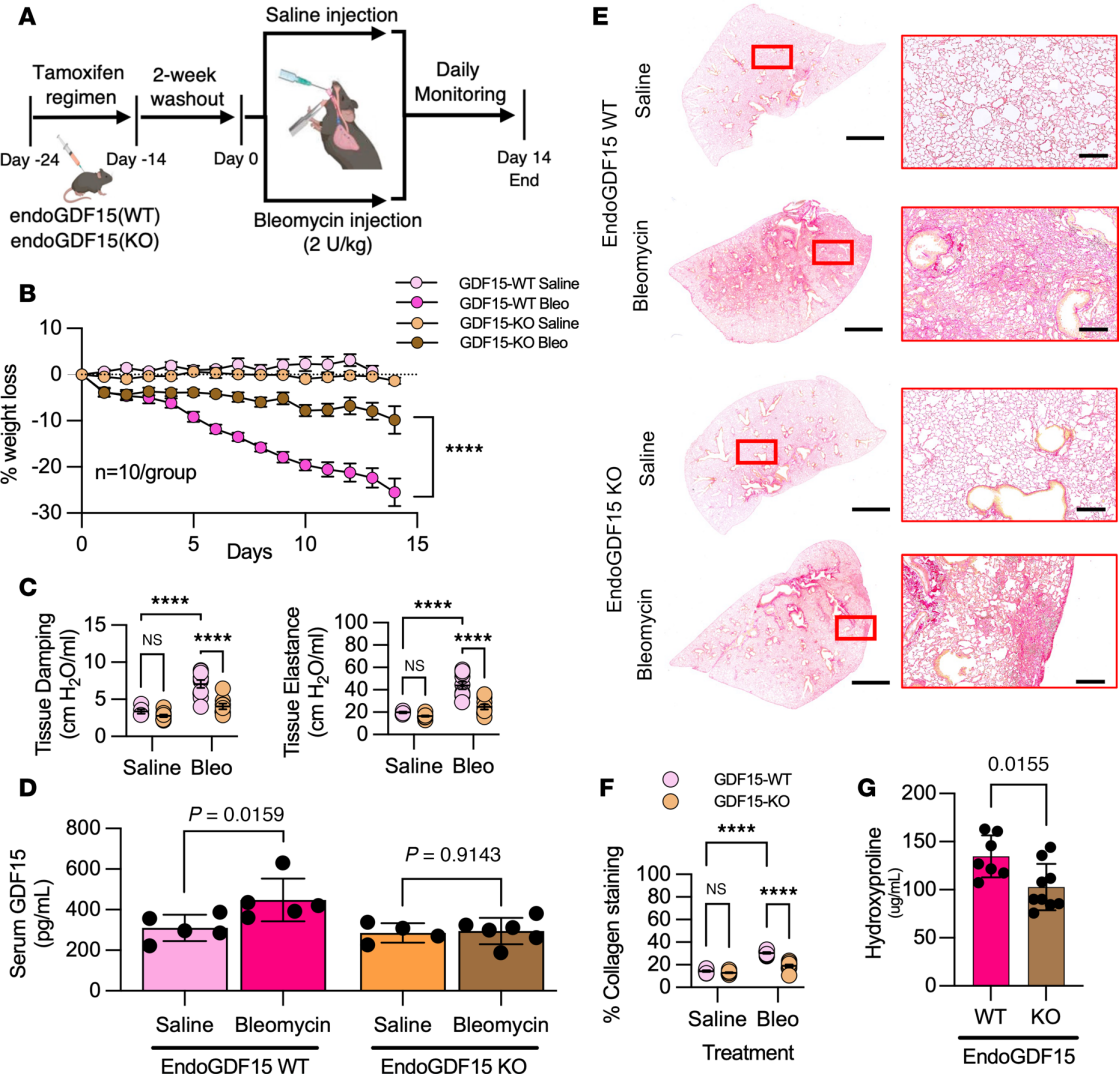
Murine endothelial KO of *Gdf15* is protective. (**A**) Schematic depicting experimental timeline. Endothelial *Gdf15-*KO was performed over 2 cohorts of mice. (**B**) Percent weight loss from baseline across EndoGDF15 WT and EndoGDF15-KO mice treated with either intratracheal saline or bleomycin (Day 0). *n* = 5 per group (EndoGDF15 WT Saline), *n* = 10 per group otherwise. (**C**) Pulmonary function data acquired from EndoGDF15 WT and EndoGDF15-KO mice at day 14. (**D**) Serum GDF15 levels from EndoGDF15 WT and EndoGDF15-KO mice, collected at day 14, measured using a Luminex assay. (**E**) Representative picrosirius red staining of murine histologic sections from EndoGDF15 WT (*n* = 10) and EndoGDF15-KO (*n* = 10) mice, treated with either intratracheal saline (*n* = 5 per group) or bleomycin (*n* = 5 per group). Scale bar in whole lung: 2 mm. Scale bar in zoom: 200 μm. (**F**) Quantitative analysis of trichrome-stained histologic murine lung sections from EndoGDF15 WT (*n* = 18) and EndoGDF15 KO (*n* = 18) mice, treated with either intratracheal saline or bleomycin. (**G**) Quantitative analysis of hydroxyproline in lung tissue from bleomycin-treated EndoGDF15 WT and EndoGDF15-KO mice. One-way repeated measures ANOVA (**B**), 2-way ANOVA with post hoc Holm-Šídák’s multiple comparison test (**C**), 2-way ANOVA as well as unpaired 2-tailed *t* test between 2 groups (**D**), 2-way ANOVA (**F**), Welch’s *t* test (**G**). ****P < 0.0001.

To investigate the potential endothelial-specific role of GDF15 in ILD, we generated an inducible endothelial KO mouse model. *Gdf15 (fl/fl)* mice were crossed with Cdh5(PAC)-Cre (ERT2) transgenic mice to produce a tamoxifen-inducible endothelial-specific *Gdf15*-KO strain, hereafter referred to as EndoGDF15 KO. Cdh5(PAC)-Cre (ERT2) crossed with *Gdf15* (WT/WT) served as controls, hereafter denoted as EndoGDF15 WT. All mice, regardless of genotype, received tamoxifen for 14 days followed by a 2-week washout period prior to intratracheal instillation of either saline or bleomycin (Figure 3A). KO efficiency was confirmed by RNAscope for *Gdf15* mRNA, which revealed markedly reduced endothelial *Gdf15* signal in EndoGDF15-KO lungs compared with EndoGDF15 WT (Supplemental Figure 3A). Further validation was obtained by endothelial cell isolation (*n* = 5 per group) with subsequent qPCR, demonstrating significantly reduced *Gdf15* transcript levels in EndoGDF15-KO mice with comparable *Pecam-1* expression (Supplemental Figure 3, B and C), dually confirming endothelial-specific deletion of *Gdf15*.

Endothelial-specific *Gdf15* deletion conferred protection against bleomycin-induced lung injury, as evidenced by reduced weight loss in EndoGDF15 KO as compared with EndoGDF15 WT controls (Figure 3B). Moreover, PFT demonstrates attenuation of bleomycin-associated increases in tissue damping and elastance in the EndoGDF15-KO cohort (Figure 3C). Serum GDF15 levels were elevated in bleomycin-treated EndoGDF15 WT mice relative to saline controls. This relative increase in circulating GDF15 was absent in EndoGDF15-KO mice (Figure 3D), indicating that endothelial cells contribute substantially to circulating GDF15 levels under fibrotic stress. Picrosirius red stain (Figure 3E), in parallel with quantification of collagen via trichrome stain (Figure 3F) and hydroxyproline assay (Figure 3G), revealed reduced collagen in bleomycin-treated EndoGDF15-KO mice compared with WT counterparts, further supporting a profibrotic role for endothelial-derived GDF15.

### GDF15 knockdown in pulmonary microvascular endothelial cells is associated with changes in junctional proteins.

**Figure 4:**
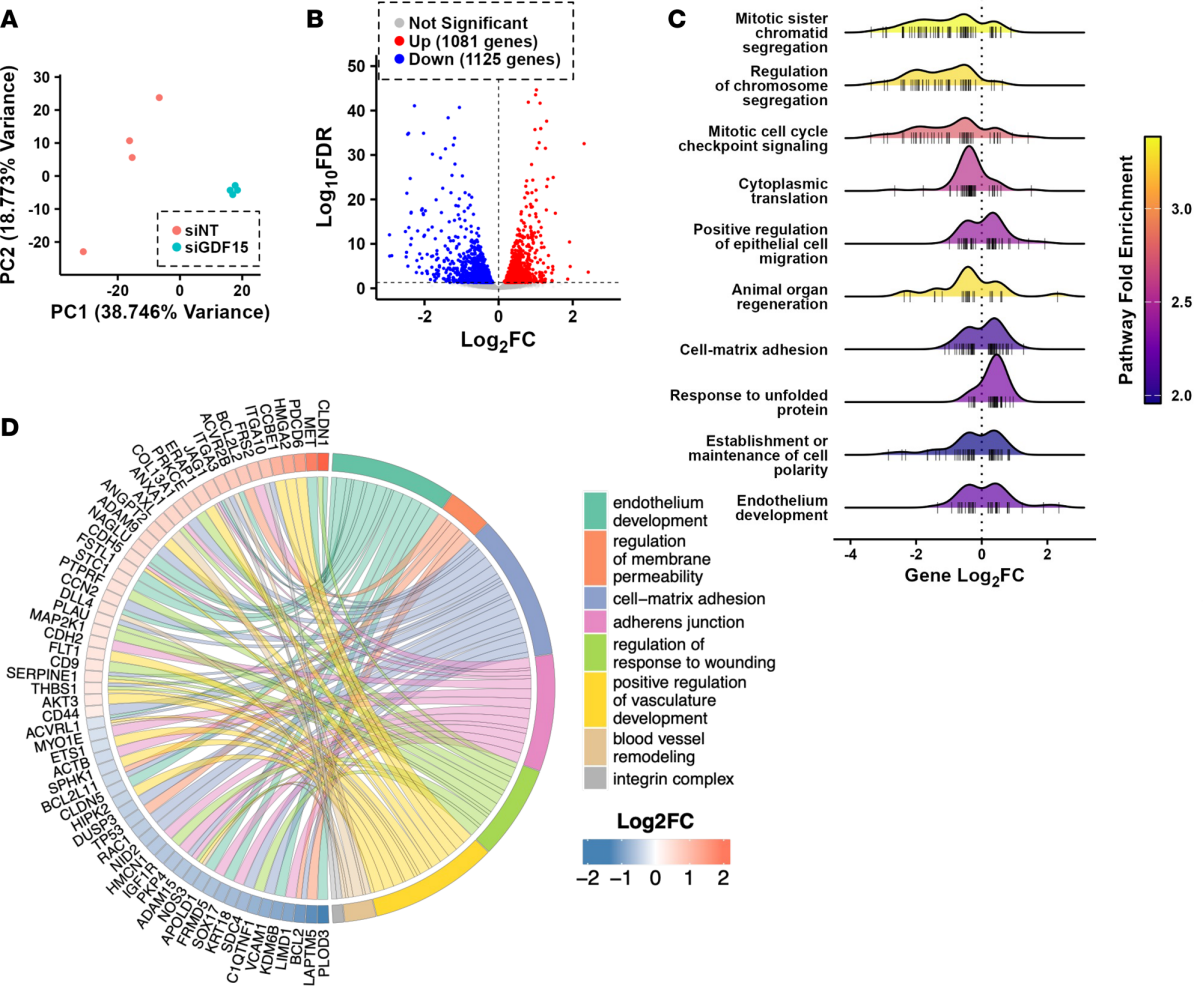
RNA-seq reveals upregulation of junctional proteins with *GDF15* knockdown. (**A**) Based on the log-transformed count per million reads (CPM) values, we selected the top 1,000 genes with the highest variance, which were used for principal component analysis. The samples are shown as dots according to their positions on the first 2 principal components (PC1/PC2). The samples are colored by their groups. (**B**) The volcano plot shows how gene expression is altered by *GDF15* silencing. Genes are shown as dots based on their log_2_ fold change (log_2_FC) and –log_10_FDR comparing siGDF15 to siNT. Significant genes (FDR < 0.05) are colored in red (upregulated) or blue (downregulated). (**C**) The top 10 enrichment Gene Ontology biological processes, ranked by their FDR, are shown as density plots. The height of the density plot indicates the number of genes with the indicated log_2_ fold change (log_2_FC). Individual genes are shown as bars under the density plot. The color of the density plot represents the fold enrichment in the over-representation test. (**D**) Significant pathways of interest (*P* < 0.05) are selected. The most significant genes (FDR < 10^–4^) involved in these pathways are linked to the pathways in a chord plot. The genes are ranked in descending order based on log2FC (siGDF15 over siNT), which is indicated by the color bar next to the gene name.

To evaluate the effects of GDF15 in endothelial cells in vitro, *GDF15* was silenced in cultured human pulmonary microvascular endothelial cells (HPMVECs), resulting in a ~87% decrease in RNA expression (Supplemental Figure 4A) and ~64% decrease in secreted protein in the media (Supplemental Figure 4B). RNA was isolated and sent for bulk RNA-seq. *GDF15* siRNA resulted in consistent and distinct transcriptomic changes, as indicated by the principal component analysis (Figure 4A). Differential gene expression analysis showed 1,081 up- and 1,125 downregulated genes (FDR < 0.05) due to the loss of *GDF15* (Figure 4B). Pathway enrichment analysis revealed the 10 most significant (ranked by FDR) Gene Ontology pathways in differentially expressed genes (Figure 4C). Interestingly, genes involved in cell division, cell cycle progression, and cytoplasmic translation were predominantly downregulated by *GDF15* silencing (Figure 4C) with no change in cell viability (Supplemental Figure 4C), suggesting GDF15 deficiency results in a quiescent phenotype. Similarly, cell migration, cell-matrix adhesion, and cell polarity regulation are also among the top affected Gene Ontology biological processes. Although genes involved in these pathways did not show a consensual direction of change, they suggested that GDF15 deficiency regulates cell adhesion and cytoskeletal organization. Therefore, we examined all significant pathways (*P* < 0.05) and focused on those related to cell-cell/cell-matrix adhesion and the cytoskeleton (Figure 4D). Specifically, genes that play a role in barrier function and whose expression was upregulated with *GDF15* silencing include *ITGA3*, *ITGA10*, *CDH5*, *CDH2*, *JAG1*, *DLL4*, *FLT1*, and most abundantly *CLDN1*. These data suggest a role for GDF15 in barrier integrity.

### GDF15 knockdown preserves endothelial cell monolayer resistance following thrombin challenge.

**Figure 5:**
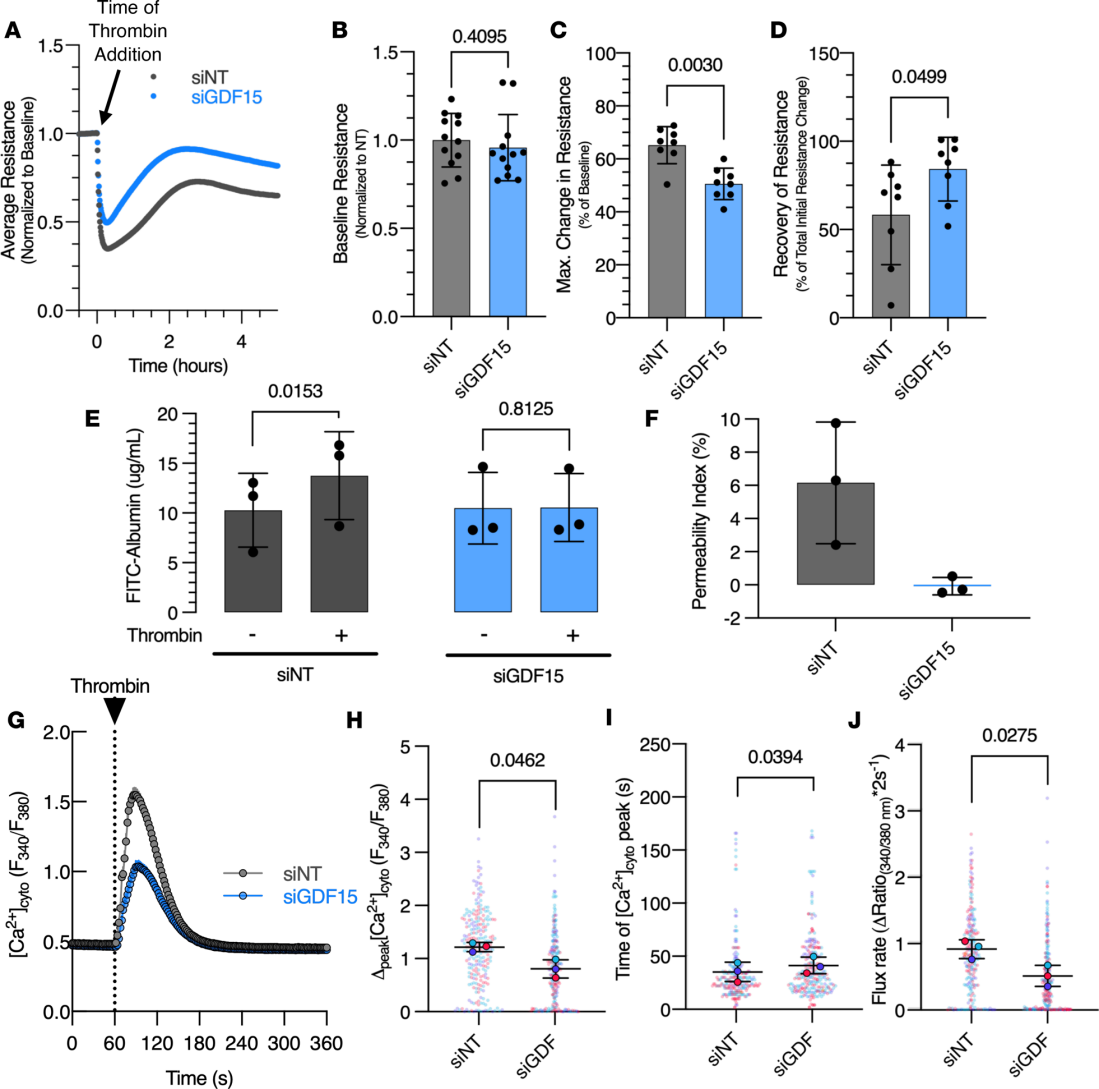
*GDF15* Knockdown is associated with decreased change in permeability in response to insult, at least in part due to decreased cytosolic Ca^2+^. (**A**–**D**) Electric cell-substrate impedance sensing (ECIS) on HPMVECs. Cells underwent knockdown with siRNA for 48 hours followed by serum starvation for 4 hours prior to the initiation of ECIS. (**A**) Average resistance over time, normalized to baseline resistance. Each run was performed in quadruplicate; each condition is an average of 8 runs. Thrombin treatment at time = 0 seconds. (**B**) Baseline resistance of siNT- and siGDF15-HPMVECs, normalized to siNT (*n* = 12 per condition). (**C**) Maximum change in resistance following thrombin treatment, presented as a percentage of baseline resistance (*n* = 8 per condition). (**D**) Recovery of resistance following the maximum drop in resistance with thrombin treatment, presented as a percentage of total initial resistance drop (*n* = 8 per condition). (**E** and **F**) Thrombin-induced FITC-albumin diffusion across a monolayer of siNT- and siGDF15-HPMVECs. (**E**) FITC-albumin concentration in plate well at 300 minutes. (**F**) Permeability index = [X – c] / [m – c] × 100, where X, c, and m are the concentration of FITC-albumin in the wells below treated cells, untreated cells, and acellular membranes, respectively. (**G**–**J**) Epifluorescence Ca^2+^ imaging analysis of HPMVECs 48 hours following siRNA-mediated *GDF15* knockdown. (**G**) Representative traces of cytosolic Ca^2+^ levels in HPMVECs over time, showing responses to thrombin stimulation. Sample sizes: NT, *n* = 244 cells; siGDF15, *n* = 261 cells. Data were pooled from 3 independent experiments. (**H**) Maximum cytosolic Ca^2+^ levels following thrombin stimulation. (**I**) Time to reach maximum Ca^2+^ response relative to thrombin application. (**J**) Rate of change in Ca^2+^ level following thrombin stimulation, calculated as the slope of the initial response. Data were analyzed using the Mann-Whitney *U* test (**B**–**D** and **H**–**J**); Welch’s *t* test (**E**). Data are presented as mean ± SEM unless otherwise indicated.

Given significant alteration of several junctional proteins with endothelial *GDF15* knockdown, we hypothesized that GDF15 has a role in barrier integrity. siNT- and siGDF15-HPMVECs were plated onto an Electrical Cell-Substrate Impedance Sensing (ECIS) chip. Once a monolayer was established, the cells were treated with thrombin 1 U/mL and changes in resistance were measured at a frequency of 4,000 Hz (Figure 5A). Baseline resistance was similar between siNT- and siGDF15- HPMVECs (Figure 5B, *n* = 12). Upon thrombin stimulation, cells treated with siNT demonstrated an approximate 65% drop in transendothelial resistance, in comparison with siGDF15-HPMVECs, which demonstrated a significantly smaller drop in resistance (Figure 5C, *n* = 8). Furthermore, siGDF15-HPMVECs recovered a larger proportion of their initial drop in resistance (Figure 5D, *n* = 8). These experiments were repeated with treatment of the siRNA-treated monolayer with 5 ng/mL rGDF15 versus vehicle for 6 hours prior to thrombin application, and we found no difference with rGDF15 pretreatment (Supplemental Figure 5A). Of note, resistance measured at lower frequencies (e.g., 4,000 Hz) are sensitive to changes in the paracellular pathway and thus serve as a representation of monolayer barrier function, whereas higher frequencies (e.g., 64,000 Hz) are sensitive to confluency and, as such, function to evaluate cell attachment and/or proliferation (27). In efforts to dually confirm permeability effects, we next pursued transwell experiments, consisting of fluorescein-isothiocyanate–albumin (FITC-albumin) application ± thrombin to a monolayer of siRNA-treated HPMVECs. We found that a thrombin-treated monolayer of siNT-HPMVECs demonstrated increased permeability to FITC-albumin relative to the untreated monolayer, a difference absent in siGDF15-HPMVECs (Figure 5E). Permeability indices for siNT- and siGDF15-HPMVECs can be found in Figure 5F, underscoring a difference in permeability across the monolayer. These data suggest that endothelial GDF15 modulates vascular permeability, likely via an intracellular mechanism given that pretreatment with rGDF15 did not alter findings.

### HPMVECs with GDF15 deficit demonstrate a blunted cytosolic Ca^2+^ response to thrombin.

Given that our RNA-seq analysis highlighted multiple genes involved in cell adhesion and that in vitro experiments demonstrated altered thrombin-induced permeability in siGDF15-HPMVECs, we tested whether Ca^2+^ signaling was affected. In the absence of extracellular Ca^2+^, siGDF15-HPMVECs exhibited a reduced thrombin-induced Ca^2+^ response, characterized by a lower peak [Ca^2+^] (Figure 5, G and H), delayed thrombin-induced Ca^2+^-release kinetics (Figure 5I), and slower Ca^2+^ flux kinetics (Figure 5J), as well as fewer cells stimulated (Supplemental Figure 5B). Resting baseline cytosolic Ca^2+^ level was comparable between siNT- and siGDF15-HPMVECs, and the resting endoplasmic reticulum–store (ER-store) content unaltered in siGDF15-HPMVECs (Supplemental Figures 5, C and D, respectively). Given that the inositol triphosphate (IP3) receptors (IP3Rs) are critical for Ca^2+^ mobilization from the ER, the 3 IP3R isotypes were queried in siNT- and siGDF15-HPMVECs. *IP3R1* mRNA is significantly decreased in siGDF15-HPMVECs relative to siNT, while the mRNA levels of the other 2 isotypes remain comparable (Supplemental Figure 5E). These data suggest that the role of endothelial GDF15 in vascular permeability is via regulation of Ca^2+^ signaling.

### Endothelial-specific deletion of GDF15 in mice may confer protection against bleomycin-induced fibrosis via altered permeability.

Given that our cell-based experiments suggested a potential role for GDF15 in vascular permeability, we pursued an in vivo assessment of vascular leak utilizing Evans Blue dye (EBD) as a tracer. EndoGDF15 WT and EndoGDF15-KO mice underwent treatment with either saline or bleomycin (day 0), with subsequent injection of EBD via tail vein and sacrifice with organ harvest (day 7). EBD was quantified in bronchoalveolar lavage fluid (BALF) and in the murine lungs. While a significant increase in EBD between saline- and bleomycin-treated EndoGDF15 WT mice BALF is absent, the BALF of EndoGDF15-KO mice treated with bleomycin have significantly less EBD than those who received saline (Supplemental Figure 6A), suggesting a bolstered barrier in response to insult. Similarly, we saw increased EBD in the lung tissue of bleomycin-treated (relative to saline-treated) EndoGDF15 WT mice, whereas this significant increase in EBD was lacking in EndoGDF15-KO mice (Supplemental Figure 6B). These data would suggest that there is less EBD accumulation in the lungs of EndoGDF15-KO mice, supporting possible altered permeability as a mechanism of protection in these animals.

## Discussion

Pulmonary fibrosis is often associated with PH, which can manifest disproportionately to the extent of fibrotic remodeling, implicating a potential vascular-driven mechanism. We show that GDF15 is elevated across multiple fibrotic lung disease subtypes and correlates with markers of right heart strain, but not with pulmonary function indices, suggesting an endothelial-specific role. Thus, we developed an endothelial cell–specific *Gdf15-*KO mouse model and show that these mice are protected from bleomycin-induced lung injury, characterized by preserved pulmonary function and reduced fibrotic remodeling. Transcriptomic analysis of *GDF15*-deficient HPMVEC revealed dysregulation of genes involved in barrier integrity, and functional assays demonstrated mitigation of thrombin-induced barrier disruption and dampened cytosolic Ca^2+^ responses with *GDF15* knockdown. These findings identify, for the first time to our knowledge, a molecular link between vascular permeability and endothelial GDF15, shown here to be a modifier of lung injury and fibrosis.

The micropathology of pulmonary fibrotic disease is characterized not only by the loss of repair function (or “senescence”) and epithelial-mesenchymal transition (28, 29) but also by vascular remodeling (30), emphasizing an undefined role of the vasculature in fibrosis. As presented by Ghigna et al., there is a subgroup of patients with IPF in whom their PH is disproportionate to interstitial involvement, suggesting presence of exaggerated vascular remodeling (31). Notably, PH-complicated IPF carries a significantly higher mortality than IPF alone (8). Of patients with IPF listed for lung transplantation in the United Network for Organ Sharing (UNOS) registry between 2004 and 2005, the mean pulmonary arterial pressure (mPAP) did not correlate with FVC (6); histologic examination revealed medial thickening and intimal proliferation in vessels in, importantly, relatively nonfibrotic areas. Leuchte et al. examined a group of pulmonary fibrosis patients and, while a correlation between BNP and pulmonary vascular resistance and/or mPAP was identified, significant correlation between BNP and lung function parameters was absent (32). These prior data strongly support that there is a primary vascular process occurring alongside the parenchymal fibrotic process in ILD. Furthermore, endothelial dysfunction and remodeling has been shown to enhance fibrogenesis in vitro (33), underscoring potential “vascular-parenchymal crosstalk” in the lung.

The role of GDF15 in pulmonary fibrosis is supported by its significant elevation in lung tissue, BALF, and plasma of bleomycin-treated mice (34), as well as its upregulation in IPF lung-derived extracellular matrix (20). While GDF15 neutralization inhibited bleomycin-induced fibrosis in vivo (20), the mechanism by which GDF15 participates in pulmonary fibrosis remains obscured. Moreover, the majority of research heretofore published regarding pulmonary GDF15 is in the alveolar epithelium, both in pulmonary fibrosis (10, 20, 34) and in response to infection (35), concluding that the epithelium is the main source and mediator of GDF15 in the lung. The only high-affinity receptor for GDF15 identified to date is GDNF Family Receptor Alpha-Like (GFRAL) (19, 36), located predominantly in the area postrema and nucleus of the solitary tract of the brainstem (37) with minimal to absent expression elsewhere. A member of the TGFb superfamily, GDF15 can signal through TGFb receptors (16, 38, 39), as well as through MAPK, PI3K/AKT, and STAT3 pathways (40), with myriad documented effects outside of the neuraxis (41–44). Given its documented ability to signal in the absence of GFRAL, it is plausible that GDF15 may have a primary role in the vasculature. While GDF15 is a secreted protein (45), the endothelium has not been examined as a potential contributor to pulmonary GDF15 production.

Our findings reiterate an association between circulating GDF15 levels and multiple biomarkers of right heart strain in a cohort of patients with ILD of diverse etiologies, characterized predominantly by pulmonary fibrosis. Elevated GDF15 levels significantly associate with elevated BNP, ePASP, and TRV values in our ILD cohort, with a significant correlation noted between GDF15 and BNP (Figure 1, A–E). Notably, this aligns with prior published data: GDF15 has been shown to correlate with biomarkers of right ventricular dysfunction (25). Also of note, the assay used to measure circulating human GDF15 levels is sensitive to a common polymorphism in humans, potentially even underestimating true GDF15 levels (46). We found no correlation between GDF15 levels and PFT indices within our cohort (Figures 1, F and G). In prior research, GDF15 correlates inversely with diffusing capacity of the lungs for carbon monoxide (DLCO) (10, 47), but notably this relationship does not distinguish between fibrotic disease and microvascular pathology. There are no prior data demonstrating GDF15 correlation with FEV1 or FVC, which are unique measures of pulmonary function specifically representing parenchymal fibrotic disease. Zhang et al. did note a correlation between GDF15 and change in FVC in 1 of 3 cohorts with IPF, but did not find a correlation between GDF15 and absolute FVC in any cohort (10).

Furthermore, histologic specimens from patients with ILD demonstrate microvascular remodeling, characterized by increased microvessel density as well as increased microvessel wall area, relative to controls (Figure 2, A–D). Notably, a strong negative correlation is identified between GDF15 levels and microvessel density (Figure 2D, *r* = –0.5939). While there is not sufficient data heretofore to determine whether an absolute increase or decrease in microvessel density occurs in ILD, current data do suggest that the presence of vascular heterogeneity itself supports the presence of an active or pathologic microvasculature. Ebina et al. found heterogeneity of vascular remodeling across 7 patients with IPF, noting high vascularity in areas of minimal fibrosis and low vascularity in areas that were severely fibrotic (48). Patel et al. examined lung specimens from patients with IPF and PH, observing medial thickening and intimal proliferation even in minimally fibrotic regions (6). Colombat et al. surveyed specimens of 26 patients with IPF and found venous and arterial wall thickening with severe luminal narrowing consistently present in each patient in densely fibrotic areas (49). Therefore, there is little consensus regarding microvessel count, density, or increased vessel wall area in ILD, but the mere presence of abnormality relative to control specimens in our cohort supports the presence of vascular remodeling in these patients, all of whom also have an elevated GDF15 level (Supplemental Figure 2B). These data suggest that at least part of GDF15’s effect in pulmonary fibrotic disease may be at the endothelium, rather than exclusively at the alveolar epithelium as previously thought.

We show here that endothelial-specific GDF15 KO confers protection against bleomycin-induced pulmonary fibrosis in mice, manifesting as decreased weight loss (Figure 3B) and blunted increase in their tissue damping and tissue elastance, both of which are reflective of pressures required to overcome increased resistance to airflow in the setting of fibrosis (Figure 3C). These data highlight a profibrotic role for endothelial GDF15. Remarkably, the relative increase in serum GDF15 concentrations seen in bleomycin-treated EndoGDF15 WT mice is absent in the EndoGDF15-KO mice (Figure 3F), underscoring that a substantial portion of circulating GDF15 originates from the endothelium under conditions of stress. Our functional and serologic data align with our histologic findings, demonstrating a paucity of collagen fiber staining in bleomycin-treated EndoGDF15-KO mice, relative to similarly treated EndoGDF15 WT mice (Figure 3, E–G). These data further support an active and seemingly novel role for GDF15 at the endothelium in fibrotic disease, and a potential innovative therapeutic avenue.

Potential mechanisms by which endothelial *Gdf15*-KO facilitated protection from bleomycin-induced lung injury were elucidated with bulk RNA-seq. An enrichment analysis revealed a predominant downregulation of genes involved in cell division and cytoplasmic translation, signifying a quiescent phenotype, given no difference in cell viability (Supplemental Figure 4C). This is alongside an upregulation of genes associated with cell migration, cell-matrix adhesion, and cell polarity regulation, prioritizing altered endothelial barrier integrity as the most plausible mechanism by which GDF15 influences fibrotic remodeling. Future work defining the molecular mechanism and transcription factor contribution to these gene changes is warranted and will be the subject of future investigation.

Hypoxia, a well-established clinical sequela of pulmonary fibrosis, induces altered endothelial barrier function via changes in actin fiber formation, thereby increasing transendothelial permeability often with the development of subendothelial edema (50). We hypothesized that the mechanism of protection afforded EndoGDF15-KO mice was secondary to altered microvascular junctional integrity, possibly facilitating a change in inflammatory infiltration into the extracellular milieu. We utilized electric cell-substrate impedance sensing (ECIS) (Figure 5, A–D), as well as FITC-albumin translocation (Figure 5, E and F), across a monolayer of HPMVECs to demonstrate that *GDF15* knockdown blunted the thrombin-induced changes in barrier integrity. Notably, we did not see a difference in baseline resistance between siNT- and siGDF15-HPMVECs (Supplemental Figure 5C), underscoring GDF15’s role in reactive modulation of permeability as opposed to maintenance of resting transendothelial permeability. An EBD in vivo assessment of “vascular leak” in EndoGDF15 WT and KO mice further supports a potential role for endothelial GDF15 in barrier function modulation, with EndoGDF15-KO mice lacking a significant increase in EBD in lung tissue upon treatment with bleomycin relative to their WT counterparts (Supplemental Figure 6). However, these data are interpreted cautiously, as there was no significant difference in lung tissue EBD between bleomycin-treated EndoGDF15 WT mice and bleomycin-treated EndoGDF15-KO mice. While it has been heretofore demonstrated that the endothelium may play an important role in pulmonary fibrotic disease progression, we believe that both the concept of GDF15 originating in the endothelium as well as its significant effect on barrier function are novel.

Endothelial barrier function is maintained largely by tight junctions and adherens junctions (51), both of which are Ca^2+^ dependent (52, 53). Thus, we postulated that altered Ca^2+^ signaling was facilitating the modified barrier function seen with *GDF15* knockdown. We utilize live-cell Ca^2+^ imaging of thrombin-treated HPMVECs to demonstrate altered calcium kinetics in siGDF15-HPMVECs (Figure 5, G–J), suggesting either a difference in Ca^2+^ handling or total cellular Ca^2+^. Thrombin primarily activates endothelial cells through protease-activated receptor 1, a G-protein-coupled receptor (GPCR) that triggers Gq protein signaling (54) upon cleavage by thrombin. This activates phospholipase C β (PLCb) (55), which hydrolyzes phosphatidylinositol 4,5-bisphosphate into inositol 1,4,5-triphosphate (IP3) and diacylglycerol. IP3 then binds IP3 receptors (IP3Rs) on the ER, causing Ca^2+^ release into the cytoplasm, an essential early step in thrombin-triggered endothelial signaling. To test whether a difference in ER Ca^2+^ stores underlay the difference in thrombin-induced cytosolic Ca^2+^ levels between siNT- and siGDF15-HPMVECs, cells were subjected to thapsigargin (a sarco/endoplasmic reticulum Ca^2+^-ATPase [SERCA] pump inhibitor) in the absence of extracellular Ca^2+^ to assess ER store Ca^2+^ content directly (Supplemental Figure 5D), demonstrating comparable total Ca^2+^ mobilization from the ER. Thus, the reduced thrombin-provoked Ca^2+^ response seen in siGDF15-HPMVECs may be attributable to a difference in the mechanism of ER Ca^2+^ release, which is mainly dependent upon IP3R activity. There are 3 IP3R isotypes (56, 57) that form homo- or heterotetramers to form a functional unit (58), and the channel’s affinity for IP3, and thus the subsequent ER Ca2+ release, is influenced by its composition (59). While there are data to suggest that IP3R1 is not the predominant isotype in the pulmonary microvasculature (60), we do see a ~25% decrease in *IP3R1* by qPCR with *GDF15* knockdown with no change in *IP3R2* or *IP3R3* (Supplemental Figure 5E). Alternatively, the blunted thrombin-induced Ca^2+^ response in siGDF15-HPMVECs could be due to changes in GPCR signaling, downstream PLC-IP3 production, or modifications in posttranslational processes that affect channel gating or ligand sensitivity. Overall, these mechanisms underscore the complex control of thrombin-induced Ca^2+^ signaling and highlight multiple potential points where GDF15 might influence endothelial Ca^2+^ dynamics, the subject of future investigation.

In summary, we demonstrate a role for GDF15 at the endothelium in pulmonary fibrotic disease and identify augmented reactive barrier function with *GDF15* knockdown that is likely due to alterations in Ca^2+^ release from the ER. While we certainly understand this to be novel from the perspective of GDF15, intracellular Ca^2+^ effects are myriad and determination of the exact mechanism will require further expansive and diverse inquiry. Moreover, the only receptor identified to date (GFRAL) is located almost exclusively in the brain, bringing to light that GDF15 must be signaling via an alternate receptor in the pulmonary microvasculature or affecting change intracellularly, an important distinction if we are to explore the potential therapeutic targeting of intrapulmonary endothelial GDF15. Given how widely expressed GDF15 is, precise tissue targeting would be of paramount importance. Key strategies to target the pulmonary endothelium have included targeting endothelial surface markers such as Intercellular Adhesion Molecule 1 (ICAM-1) (61), which can be coupled with more cutting-edge technology such as Charge Altering Releasable Transporters (CARTs), able to effectively deliver viable mRNA cargo to tissue (62). Future investigations will elucidate details regarding the mechanism by which GDF15 affects changes in Ca^2+^ signaling, clarify the avenue by which GDF15 signals outside of the neuraxis, and illuminate potential opportunities for intervention to reduce disease burden in pulmonary fibrosis.

## Methods

### Sex as a biological variable.

All mice utilized for these experiments were male; sex was not considered as a biological variable.

### Clinical cohort.

Subjects were recruited from the Simmons Center for Interstitial Lung Disease at the University of Pittsburgh Medical Center (UPMC) between January 2003 and October 2016 (*n* = 373). Members of the cohort with an ejection fraction on TTE < 40% were eliminated from our study. Demographic characteristics can be found in Supplemental Table 1. Specific diagnostics were performed according to standard criteria and current ATS guidelines. Control subjects without any self-reported history of lung disease were randomly recruited at UPMC, *n* = 72. Plasma GDF15 levels were measured with a human GDF15 Quantikine ELISA kit (R&D Systems). BNP was measured clinically, and the standard upper limit of 100 ng/mL was utilized. ePASP and TRV measurements were acquired via TTE; measurements utilized were obtained within 24 months of GDF15 level. Control versus disease was analyzed with a 2-sample independent 2-tailed *t* test for *n* > 10 per group, and with a Kolmogorov-Smirnov test for comparisons with *n* ≤ 10 per group. The relationship between 2 variables was assessed using simple linear regression or Pearson correlation. Cohort PFT was arbitrarily separated into disparate severity groupings according to the FVC (percent predicted), after excluding those patients with a FEV1/FVC ratio less than 70% (*n* = 303). PFTs utilized were performed within 12 months of GDF15 level; if there were multiple applicable tests, the one closest to the date of GDF15 level acquisition was utilized. For longitudinal follow-up data: any patients within our ILD cohort who had multiple TTEs or PFTs within a 2-year follow-up period were included, using the most historical and the most recent studies for comparison. GDF15 levels were analyzed across 8 subgroups of ILD via a Kruskal-Wallis with a post hoc Dunn’s test.

### Immunofluorescence of human lungs.

Formalin-fixed, paraffin-embedded lung tissue obtained from patients with ILD who progressed to transplant (*n* = 11) and healthy control patients (*n* = 6, obtained from organ donors not suitable for transplantation) were cut into 4 μm sections. Each section was deparaffinized in xylene and rehydrated in graded concentrations of ethanol before antigen retrieval (Target retrieval solution, citrate pH 6, Agilent, Dako) in a pressure cooker at 40°C for 20 minutes. Slides were rinsed in washing buffer (0.01M Tris, 0.15 M NaCl and 0.05% Tween 20) and incubated in 300 mM glycine for 30 minutes. Lung sections were blocked (2% BSA, 0.1% Triton, 0.1% Tween) for 1 hour at 55°C. The sections were incubated overnight in humidified conditions at 4°C with primary antibodies against SMA (Alexa Fluor 488–conjugated SMA; 1:150, Invitrogen, 53-9760-82), platelet endothelial cell adhesion molecule 1 (Alexa Fluor 647–conjugated anti-PECAM-1; 1:50, Santa Cruz, sc376764), and GDF15 (5 μg/mL, Invitrogen, 42-1700). The sections were then incubated in Alexa Fluor 750-conjugated donkey anti-rabbit (2 μg/mL, Biotium, 20298) for 1 hour at room temperature. Sections were treated with TrueView autofluorescence quenching reagent (Vector Laboratories, SP-8400-15), and counterstained with DAPI (1:10,000; Biotium, 40043). Images were taken at 20X using an IX83 Olympus microscope and analyzed using QuPath software. Histologic sections were analyzed for medial thickening; 3 vessels less than 50 μm in diameter were randomly selected per section and medial thickening was determined by subtracting lumen area from total vessel area. A depiction of how arteriole wall area was calculated can be found in Supplemental Figure 2A. Sections were analyzed for vessel count; 4 random fields were selected per section, in which total number of vessels with average diameter < 50 μm (average of 2 separate diameters measured in 2 axes per vessel) were counted, and the 4 fields’ counts per section were averaged. Control versus disease was analyzed with a 2-sample independent *t*-test.

### Animals.

The *Gdf15 (fl/fl)* mouse was a gift from Jonathan Alder, at the University of Pittsburgh (39). The *Gdf15 (fl/fl)* mice were crossed with Cdh5(PAC)-Cre (ERT2) transgenic mice, to generate an endothelial-specific KO of *Gdf15*, denoted hereafter as EndoGDF15 KO. Control mice, icdh5-Cre (+)/WT GDF15, are hereafter denoted as EndoGDF15 WT. Animals used for experiments received 2 weeks of daily tamoxifen injections (2 U/kg, in sterile USP-grade PBS) to induce KO, followed by a 2-week wait period prior to intratracheal instillation of saline versus bleomycin under moderate sedation. Mice were sacrificed for PFT, organ harvest, and terminal serum collection at day 14 after instillation of bleomycin. Utilizing the FlexiVent system, murine lung mechanics were measured by single frequency forced oscillation technique (FOT). Mice were fed standard chow throughout the duration of the experiment. Daily weights were obtained throughout the duration of the experiment. Animals were housed in approved USDA OLAW-registered and AAALAC-accredited facilities at the University of Pittsburgh, in ventilated racks with automatic water systems on a 12-hour light/12-hour dark cycle with access to a standard chow.

### Murine endothelial cell isolation.

Primary murine lung endothelial cells were obtained from 20-week-old EndoGDF15 WT and EndoGDF15-KO mice (1 isolation per mouse, *n* = 5 per genotype). Mice were perfused with 10 mL PBS prior to lung explantation, to reduce circulating RBCs in the murine lung. The bilateral lungs were then excised and mechanically minced, followed by digestion with Collagenase A (Millipore Sigma, #10103578001) at 1 mg/mL in PBS. Homogenate was transferred to a 15-mL Eppendorf tube and incubated at 37°C with agitation for 1 hour, followed by filtration through a 70 μm mesh filter and centrifugation. The pellet was resuspended and incubated with RBC lysis buffer. After centrifugation at 500 × *g*, the pellet was washed with suspension buffer (Ca^2+^ and Mg^2+^-free PBS, 2 mM EDTA, 4.5 mg/mL D-glucose). To the final suspension, 1 μg of anti–PECAM-1 antibody (BD Pharmingen, 553370) was added at 4°C for 60 minutes. Sheep anti-rat IgG Dynabeads (Invitrogen, 11035) were added to the suspension at 4°C for 30 minutes. Following incubation, the suspension was washed with suspension buffer. RNA lysis buffer was added to the final pellet, and the sample was processed as per below to isolate RNA.

### Murine histopathology.

After sacrifice, lungs were perfused with 10% neutral-buffered formalin followed by paraffin embedding. Histopathologic changes and fibrosis were evaluated using picrosirius stain and trichrome stain according to manufacturer’s protocol. Hydroxyproline was quantified using a colorimetric hydroxyproline assay (Abcam, ab222941).

### EBD assessment.

Animals received either saline or bleomycin (day 0). At day 7, the animals underwent tail vein injection of EBD (100 μL, 1% EBD in sterile PBS) with subsequent sacrifice at 60 minutes. A bronchoalveolar lavage (BAL) was performed (500 μL); absorbance was measured at 620 nm and 740 nm (for correction) over technical duplicates. Serum was collected and diluted 1:10; absorbance was measured at 620 nm over technical triplicates. The lungs were perfused with cold PBS to remove intravascular EBD, and the bilateral murine lungs were removed and placed in cold PBS. Lungs were then placed in formamide and heated at 55°C for 24 hours for EBD extraction; the samples were then centrifuged at 3,000 × *g* and supernatant was collected. Absorbance was measured at 620 nm, 740 nm (for correction), and 550 nm (hemoglobin), over technical triplicates. The lung formamide eluent was corrected for residual lung tissue and hemoglobin, and normalized to serum EBD and body weight. The manuscript by Smith et. al. was utilized for guidance (63).

### RNA scope.

Formalin-fixed, paraffin-embedded sections were managed according to manufacturer’s protocol and counterstained with DAPI (RNAscope Intro Pack for Multiplex Fluorescent Reagent Kit v2 Mm, Cat. No. 323136; RNAscope probe against Mm GDF15, Cat. No. 318521; TSA Vivid Fluorophore 650, Cat. No. 323273). Images were taken at 20X using an IX83 Olympus microscope. Images were analyzed using QuPath software.

### GDF15 expression and circulating GDF15 levels.

RNA was isolated from cells using the RNeasy kit (Qiagen, 74104). Relative *GDF15* mRNA levels were measured using the comparative Ct method of real-time quantitative PCR using a Life Tech Quant Studio 5. Primers specific for human *GDF15* (ACCAGAGCTGGGAAGATTCG, CGAGAGATACGCAGGTGCAG), human *GAPDH* (ATGACATCAAGAAGGTGGTG, CATACCAGGAAAATGAGCTTG), mouse *Gdf15* (ACTCGAACTCAGAACCAAGTC, AGACCCTGACTCAGCGA) and mouse *Gapdh* (CCTCGTCCCGTAGACAAAATG, TGTAGTTGAGGTCAATGAAGGG) were utilized with *GAPDH* serving as the housekeeping gene. Measurement of circulating GDF15 levels in murine serum was obtained via a custom 13-Plex Mouse Luminex Discovery assay (R&D Systems, LXSAMSM-13). GDF15 was measured in media of human pulmonary microvascular cells using an ELISA assay (Abcam, ab155432).

### IP3R and PECAM1 expression.

RNA was isolated as above. Relative *IP3R1* mRNA levels were measured using the comparative Ct method of real-time quantitative PCR using a Life Tech Quant Studio 5. Primers specific for human *IP3R1* (CAT TGC TGG GAA GCT AGA GAA G, GTT CCA CCA GTG ACG AAG TAA A), human *IP3R2* (CAG CTC AGG CAG AAA CTA TGT, CAG ATG AAT GAG GAC CCG TAA A), human *IP3R3* (GGG ATT ACA GAC TGC CTC TTC, CTT CTC CTT GTC CTG CTT AGT C), and human *PECAM1* (CCAGCAACATTCACAGATAAG, GAAGTACCATTTCACTTCCAG) were utilized with *GAPDH* serving as the housekeeping gene.

### Cell culture maintenance.

HPMVECs obtained from Lonza (CC-2527) were cultured in EBM-2 Basal Medium (Lonza, CC-3156) supplemented with EGM-2 SingleQuots supplements (Lonza, CC-4176) in 5% CO_2_ at 37°C and used until passage #10 for experiments.

### GDF15 knockdown.

HPMVECs were seeded and grown to 70% confluence and then treated with siRNA targeting GDF15 exon 2 (Lifetech, 4392420, s18257) or nontargeting siRNA (Lifetech, 4390843), with Lipofectamine 3000 Transfection Reagent (Thermo Fisher Scientific, L3000001). Cells were harvested for analysis, or experiment was performed, at 48 hours after transfection.

### RNA-seq.

HPMVECs were transfected with nontargeting siRNA or GDF15-targeting siRNA. Samples were collected for RNA extraction and sent to Novogene for mRNA library preparation and bulk RNA-seq. Briefly, mRNA was purified from total RNA using poly-T oligo-attached magnetic beads. After fragmentation, the first strand cDNA was synthesized using random hexamer primers, followed by the second strand cDNA synthesis using dTTP for a nondirectional library. The library was then processed for end repair, A-tailing, adapter ligation, size selection, amplification, and purification. The processed library was then sequenced on an Illumina platform. Raw FASTQ files were filtered to remove low-quality reads using fastq and aligned to the human genome using Hisat2 v2.0.5. Mapped reads were then counted using featureCounts v1.5.0-p3 on the gene level. The downstream bioinformatic analysis was performed using R 4.4.1. From the gene count data, low-count genes with less than 1 count per million reads (CPM) were removed. The filtered gene counts were used by DESeq2 1.44.0 for differential gene expression analysis. Genes with adjusted *P* (FDR) < 0.05 were considered significant. We then performed over-representation pathway analysis with ClusterProfiler 4.12.6 using differentially expressed genes and the Gene Ontology database.

### Electric cell-substrate impendence sensing.

HPMVECs underwent knockdown of *GDF15*, as above. At 24 hours after transfection, the cells were trypsinized and replated onto an 8-well chip (Applied BioPhysics, 8W10E+ PC) at 60,000 cells per well and *n* = 4 wells per group. The chip was incubated at 5% CO_2_ and 37°C for another 24 hours to allow cells to adhere. At 48 hours after transfection, the cells were placed in low-serum media (1% fetal bovine serum) and incubated for another 4 hours. The chip was then attached to electrodes and baseline resistance was measured over 30–60 minutes. Subsequently, each well was treated with thrombin (Sigma, T4393) at 1 U/mL. For experiments involving rGDF15, cells were treated with rGDF15 at 5 ng/mL for 6 hours prior to thrombin application. Resistance and capacitance were recorded throughout the duration of the study for a minimum of 18 hours at a frequency of 4,000 Hz; cells were maintained at 5% CO_2_ and 37°C throughout the duration of the study. Baseline resistance was calculated as an average of resistances over a minimum of 30 minutes prior to thrombin administration. Maximal drop in resistance following thrombin administration was quantified as the lowest resistance achieved post-thrombin, reported as a percentage of baseline resistance. Recovery of resistance was quantified as the difference between the average resistance over 0.6 minutes once resistance tracing had recovered to a steady-state following thrombin administration and the resistance nadir, reported as a percentage of total initial change in resistance. NT versus KD was analyzed with a 2-sample independent *t* test.

### Transwell experiment.

Knockdown was achieved as above in HPMVECs. Cells were trypsinized at 24 hours after transfection and replated at 75,000 cells/cm^2^ in a 24-well Corning transwell plate with 0.4 um pore size, whose membranes had been coated with fibronectin prior to plating. Cells were serum-starved in 1% FBS media for 3 hours prior to experiment initiation. Fluorescein-isothiocyanate (FITC)-albumin and thrombin were applied (time = 0) and sampling of media from the well was pursued at time = 300 minutes following thrombin application. Fluorescence was measured at excitation 495 nm/emission 519 nm. Permeability index was calculated as the difference between treated and untreated cells as a fraction of maximal possible change in permeability (defined as the difference between FITC-albumin in the well across an acellular membrane alone versus across a monolayer of untreated cells) (64).

### Calcium imaging.

Knockdown was achieved as above in HPMVECs. Cells were seeded onto 25 mm Poly-D-lysine–coated coverslips (Sigma, P6407) at subconfluence at 24 hours after transfection. Just prior to the experiment, at 48 hours after transfection, cells were incubated in extracellular medium (ECM; 140 mM NaCl, 4.9 mM KCl, 1.13 mM MgCl_2_, 10 mM HEPES, 10 mM D-glucose, 2 mM CaCl_2_, pH 7.4) and loaded with 2 μM Fura-2 AM (Invitrogen, F1221) for 20 minutes at room temperature. Following dye loading, cells were washed and transferred into fresh ECM containing either 2 mM CaCl_2_ or no added Ca^2+^. Epifluorescence Ca^2+^ imaging was performed using an Olympus IX51 inverted microscope equipped with a CoolLED pE-340fura LED illumination system (340/380 nm excitation), a dual-band dichroic and emission filter set (Chroma, 73100), and a Hamamatsu ORCA-Spark camera controlled by HCImage software. Imaging was conducted using a UV-optimized Olympus UAPO 40×/1.35 NA oil-immersion objective (UAPO/340). Intracellular Ca^2+^ levels were quantified as the ratio of Fura-2 fluorescence intensity at 340 nm and 380 nm excitation (F340/F380), representing relative changes in cytosolic Ca^2+^ concentration [Ca^2+^]_cyto_.

### WST assay.

In total, 100 mM WST-8 and 20 mM 1-methoxy-PMS was applied to a cell monolayer in a 96-well plate for 3 hours at 37°C. Absorbance was measured using a microplate reader.

### Statistics.

Data are presented as mean ± SEM unless stated otherwise. Comparisons between groups were analyzed according to the statistical test specified in the figure legend. Correlation was assessed for using either a Spearman or a Pearson correlation, as specified in the figure legend. Differences between weight loss curves were calculated using 1-way repeated-measures ANOVA. Differences between multiple groups with non-normal distributions were analyzed with a non-parametric 1-way ANOVA (e.g., Brown-Forsythe and Welch ANOVA test). For the RNA-seq analysis, *P* < 0.05 was considered significant. Data were graphed using GraphPad Prism 10.6.0 (GraphPad software).

### Study approval.

The ILD registry at the Dorothy P. and Richard P. Simmons Center for Interstitial Lung Disease was approved by the IRB at the University of Pittsburgh under study nos. STUDY20030223, STUDY1900326, and STUDY19070274. Written informed consent was obtained prior to participation. All animal procurement and procedures were reviewed and approved by the IACUC of the University of Pittsburgh (nos. 24014329, 23123873) and adhered to the NIH *Guide for the Care and Use of Laboratory Animals* (National Academies Press, 2011). Animals were housed in approved USDA OLAW-registered and AAALAC-accredited facilities at the University of Pittsburgh.

### Data availability.

Data will be available upon request to the corresponding authors. Values for all data points in graphs are reported in the Supporting Data Values file.

## Author contributions

KR devised hypotheses, conducted experiments, acquired, and analyzed the data and wrote the manuscript. ACS and OE guided research hypotheses and assisted in writing and editing the manuscript. MB, BJP, PS, MM, MK, ST, SH, and BC performed experiments. SY assisted in processing the RNA-seq data. AEB assisted in microscope image acquisition and processing. JKA provided the *Gdf15* (*fl/fl*) mouse. YZ and DJK provided access to the clinical database. YZ directed the Luminex assay and sample collections. SMN assisted in statistical analyses.

## Conflict of interest

ACS is a stockholder for Creegh Pharmaceuticals and received research funds from Bayer Pharmaceuticals.

## Funding support

This work is the result of NIH funding, in whole or in part, and is subject to the NIH Public Access Policy. Through acceptance of this federal funding, the NIH has been given a right to make the work publicly available in PubMed Central.

- NIH 5T32HL007563 (KR), R01HL172493 (OE), P50AR080612 (OE) and R35HL161177 (ACS).

## Supplementary Material

Supplemental data

Supporting data values

## Version 1 04/23/2026

In-Press Preview

## Version 2 07/08/2026

Electronic publication
