# Supplementary material for: Endothelial GDF15 deficiency enhances barrier function and mitigates pulmonary fibrosis
Source: JCI Insight. 2026 Apr 23;11(13):e200198. doi: 10.1172/jci.insight.200198 (PMC13460825; doi:10.1172/jci.insight.200198)
Supplement: Supplementary file 1 [file jciinsight-11-200198-s170.pdf]

Supplemental Table 1. ILD Cohort characteristics. Age is presented as median with range and average. Unless otherwise specified, sex is assumed male. Race is presented as total *n* and percent. ILD patients were separated by ILD subtype. Idiopathic pulmonary fibrosis (IPF), hypersensitivity pneumonitis (HP), non-specific interstitial pneumonia (NSIP), pulmonary fibrosis (PF) with microscopic angiitis.

Parameter	Control	ILD
**Age**<br>Median (range), average (in years)	51 (46-74), 60	61 (18-108), 60
**Sex**		
Female n (%)	35 (48.6)	157 (42.1)
Unknown n (%)	N/A	33 (8.8)
**Race**		
Caucasian n (%)	66 (91.7)	304 (81.5)
African American n (%)	2 (2.8)	27 (7.2)
Unknown n (%)	N/A	40 (10)
**ILD Subtype**		
IPF		172 (46.1)
HP		24 (6.4)
ILD with autoimmune features		20 (5.4)
NSIP		2 (0.5)
PF with microscopic polyangiitis		1 (0.26)
Anti-synthetase syndrome		9 (2.4)
Polymyositis/dermatomyositis		19 (5.1)
Rheumatoid-associated ILD		14 (3.8)
Sarcoidosis		61 (16.4)
Sjogren’s syndrome		1 (0.26)
Scleroderma		50 (13.4)
Total n (%)	***72 (100)***	***373 (100)***

Supplemental Table 2. ILD immunofluorescence sub-cohort demographics and disease characteristics. Demographics and disease characteristics, including hemodynamic and pulmonary function measurements, across the 11 ILD patients whose lung tissue was examined with immunofluorescence.

Patient	Sex	Race	ILD Subtype	Date of Diagnosis (mm/dd/yyyy)	Diagnosis to Death (y)	GDF15 (pg/mL)	Age at GDF15 Draw (y)	BNP (pg/mL)	Ejection Fraction on TTE (%)	ePASP on TTE (mmHg)	FVC (L, %)	Notes
1	M	C	IPF	12/31/2013	7.82	1125	49	N/A	55-60	74	2.75, 56	Pirfenidone treatment; STS; s/p DLTx 9/16/16
2	M	C	IPF	05/01/2011	*Alive*	2165	54	N/A	55-60	35	2.97, 66	Familial IPF s/p lung transplant 1/28/2015
3	F	C	IPF	01/01/2014	*Alive*	1451	61	N/A	55-60	22	2.83, 50	DLTx for ILD on 3/14/18
4	M	C	IPF	04/27/2014	8.33	1536	60	313	65-70	51	2.12, 49	DLTx 4/27/2014
5	M	C	IPF	01/01/2014	7.18	3082	63	115	55-60	44	4.16, 97	Discoid lupus; DLTx 1/2019
6	M	C	IPF	01/01/2014	7.79	1388	54	20	60-65	41	4.33, 83	STRIVE-IPF trial[x]; DLTx 7/2019
7	M	C	IPF	01/01/2014	*Unknown*	1553	65	25	65-70	51	1.96, 48	DLTx 12/2014
8	F	C	HP	01/01/2014	11.57	1207	68	18	60-65	33	1.64, 62	s/p R-SLTx 8/2016
9	M	C	IPF	09/01/2014	3.05	2158	63	624	55-60	44	3.01, 66	Tracheostomy x2 at 6 y/o; s/p DLTx 7/14/15
10	F	C	IPF	11/01/2007	*Alive*	1259	66	36	55-60	46	1.86, 63	DLTx 10/2017
11	M	C	RA-ILD	*Unknown*	*Unknown*	2198	25	>5000	55-60	39	2.22, 36	s/p L-SLTx 8/2007

Sex: male (M), female (F). ILD Subtype: idiopathic pulmonary fibrosis (IPF), Rheumatoid-related interstitial lung disease (RA-ILD). Notes: short telomere syndrome (STS), underwent a double lung transplant (s/p DLTx), underwent a right- or left-sided single lung transplant (s/p R-SLTx, s/p L-SLTx). References: (x) Kulkarni T, Criner GJ, Kass DJ, Rosas IO, Scholand MB, Dilling DF, Summer R, Duncan SR. Design of the STRIVE-IPF Trial- Study of Therapeutic Plasma Exchange, Rituximab, and Intravenous Immunoglobulin for Acute Exacerbations of Idiopathic Pulmonary Fibrosis. Res Sq [Preprint]. 2024 Feb 28:rs.3.rs-3962419.

A

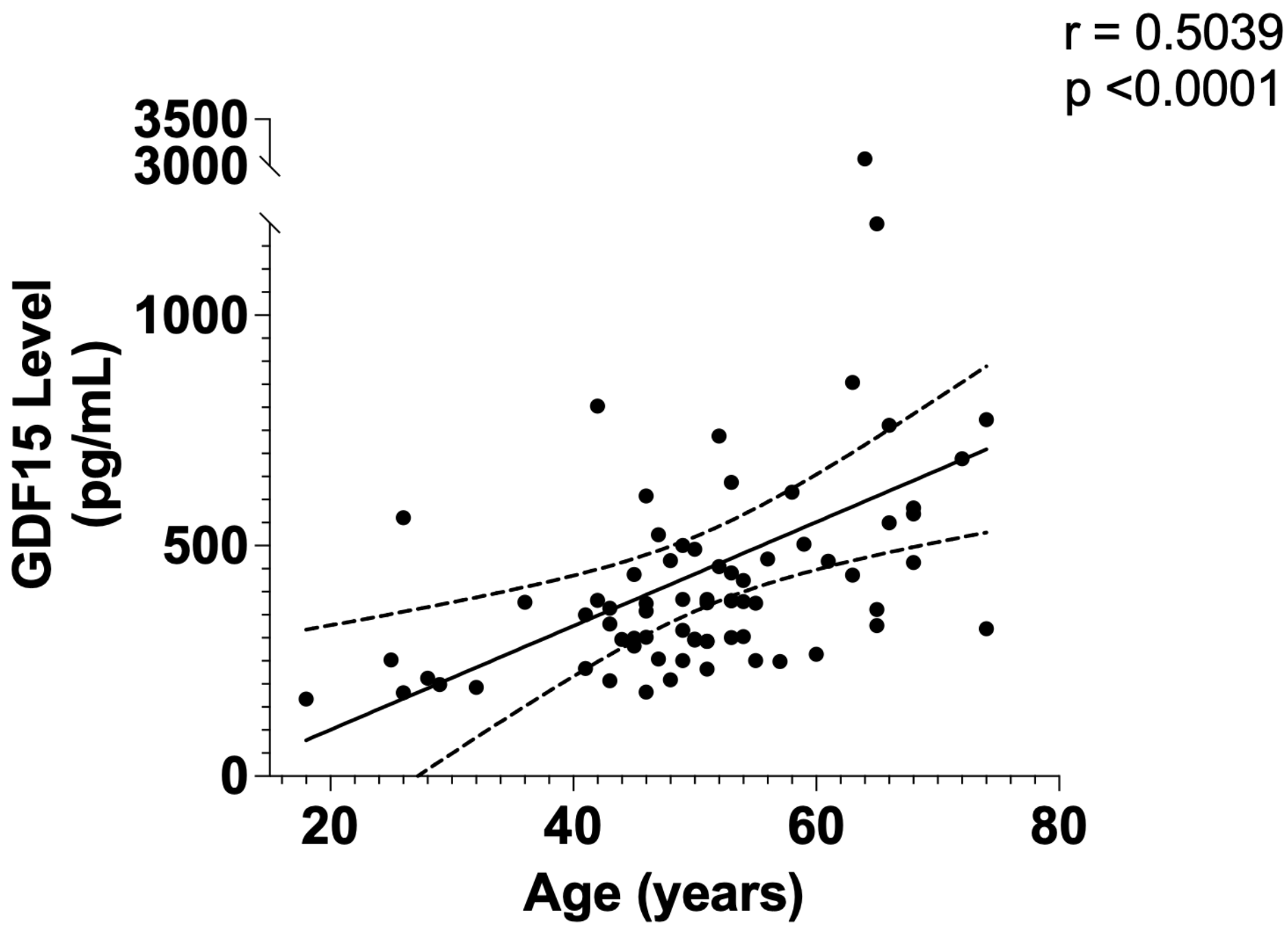

Supplemental Figure 1A. Positive correlation between log-transformed GDF15 levels and age in the control cohort. Statistical analysis: Spearman correlation.

B

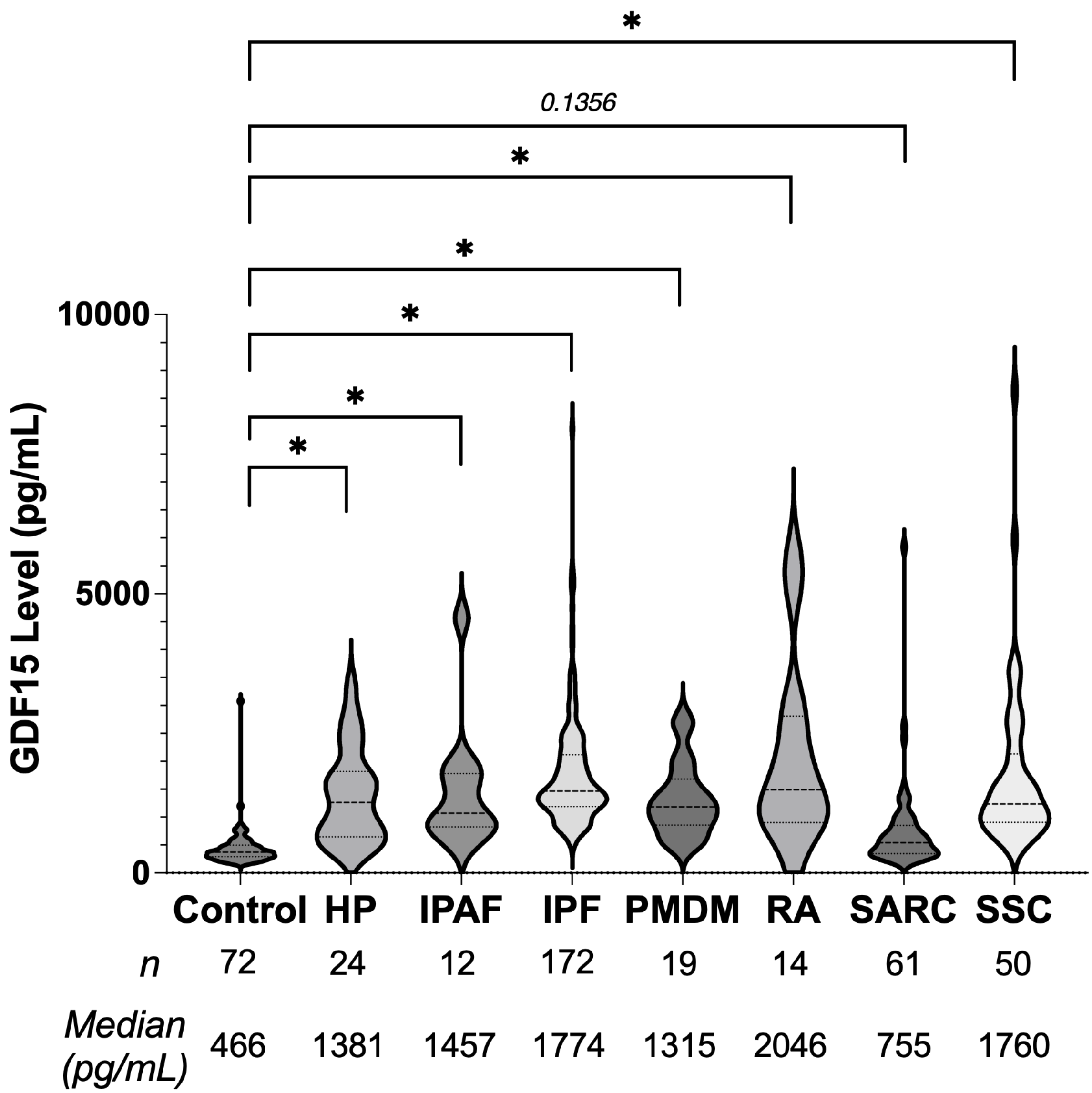

Supplemental Figure 1B. GDF15 levels per subtype of ILD. *p<0.0001. Statistical analysis: Kruskal-Wallis with a post-hoc Dunn's test.

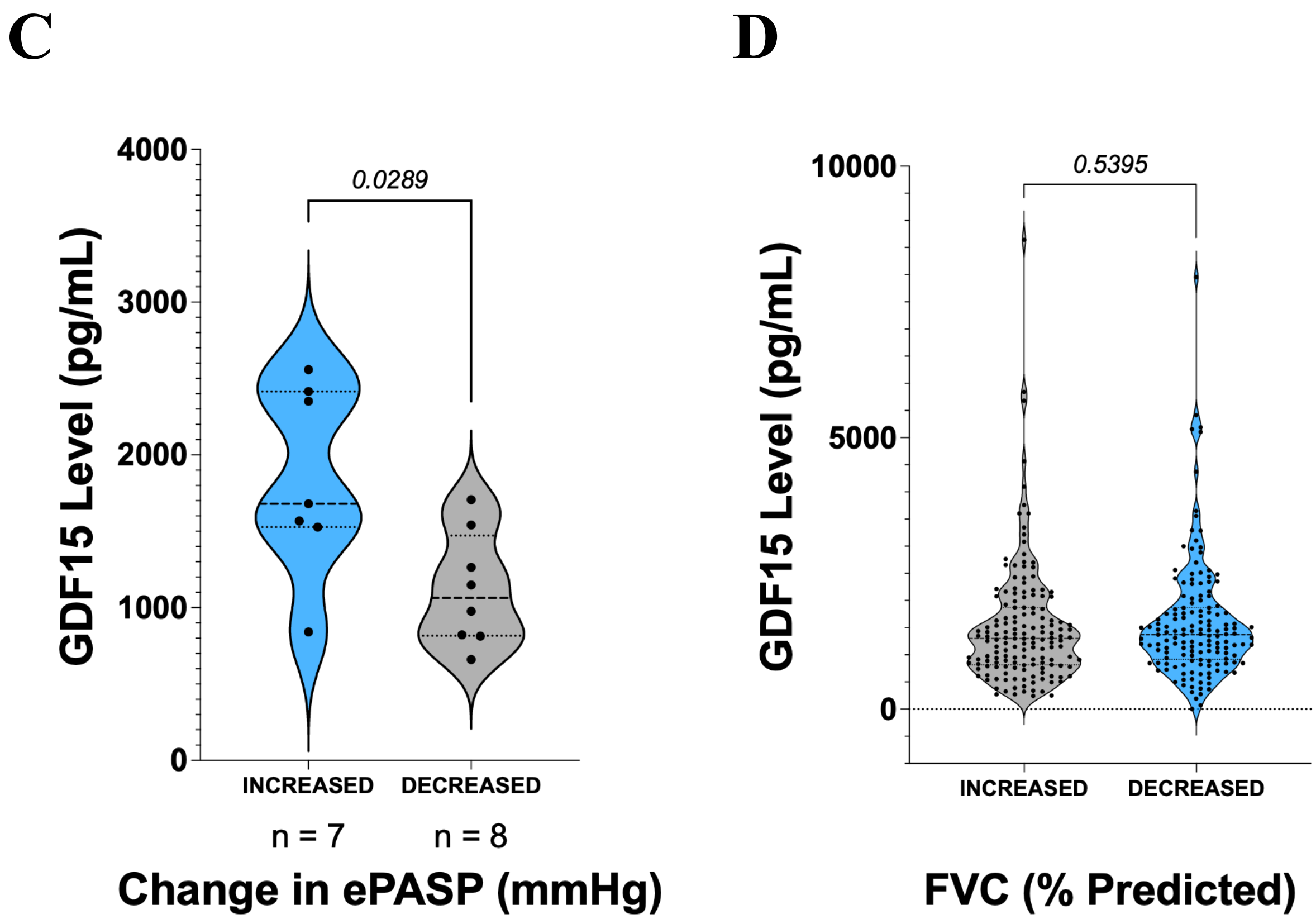

Supplemental Figure 1C. Relationship between GDF15 level and change in estimated pulmonary arterial systolic pressure on serial echocardiogram, in ILD patients. Statistical analysis: Mann-Whitney test.

Supplemental Figure 1D. Relationship between GDF15 level and change in forced vital capacity on serial testing, in ILD patients. Statistical analysis: Welch's t-test.

E

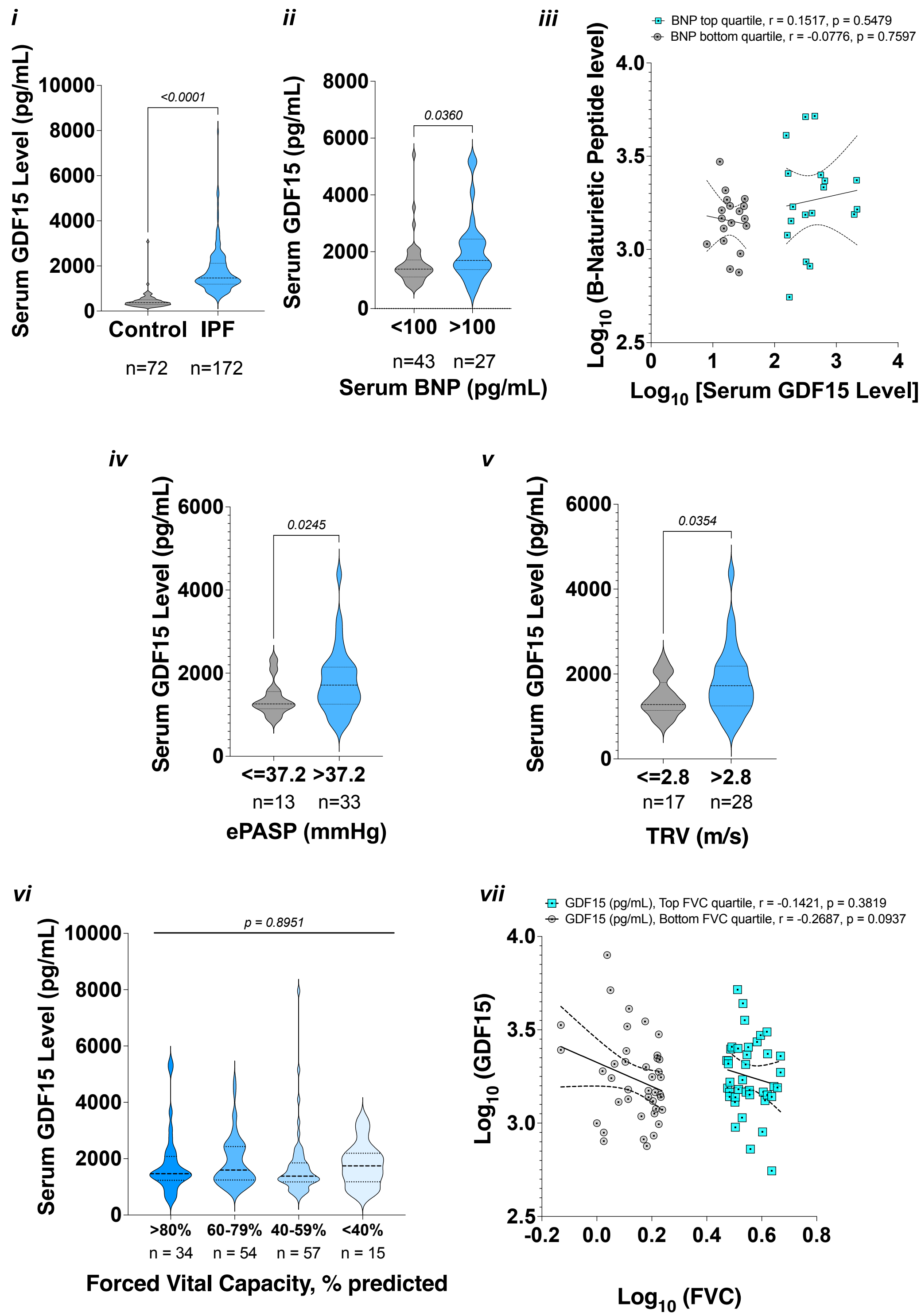

Supplemental Figure 1E. Echocardiographic data, pulmonary function testing, and serum levels of GDF15 and BNP across the sub-cohort of IPF patients. Statistical analysis: unpaired t-test (i,ii), simple linear regression (iii, vii), Welch's t-test (iv, v) Brown-Forsythe ANOVA (vi).

A

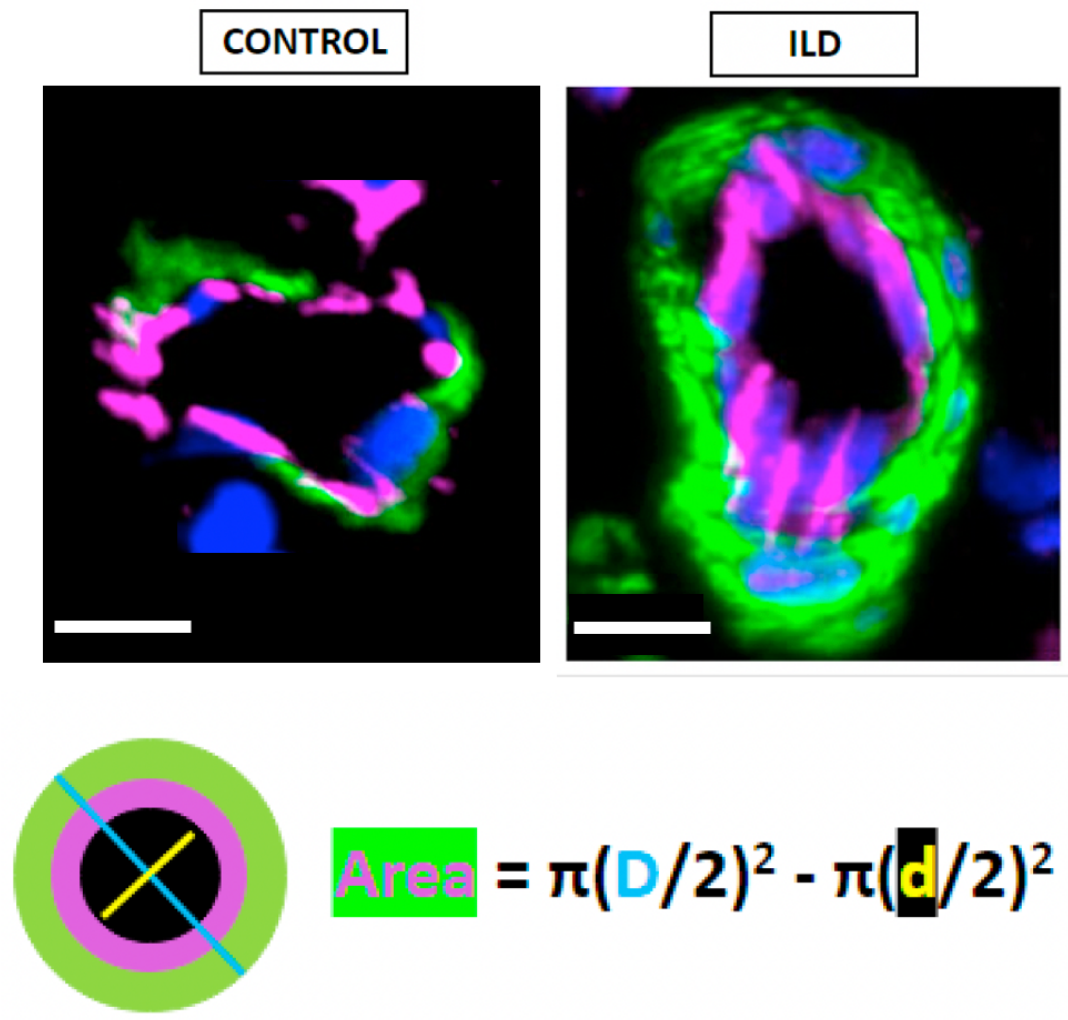

Supplemental Figure 2A. Method of measurement and calculation of microvessel wall area of vessels whose outer diameter measured < 50 um, in human histologic lung specimens. Scale bar = 10 um.

B

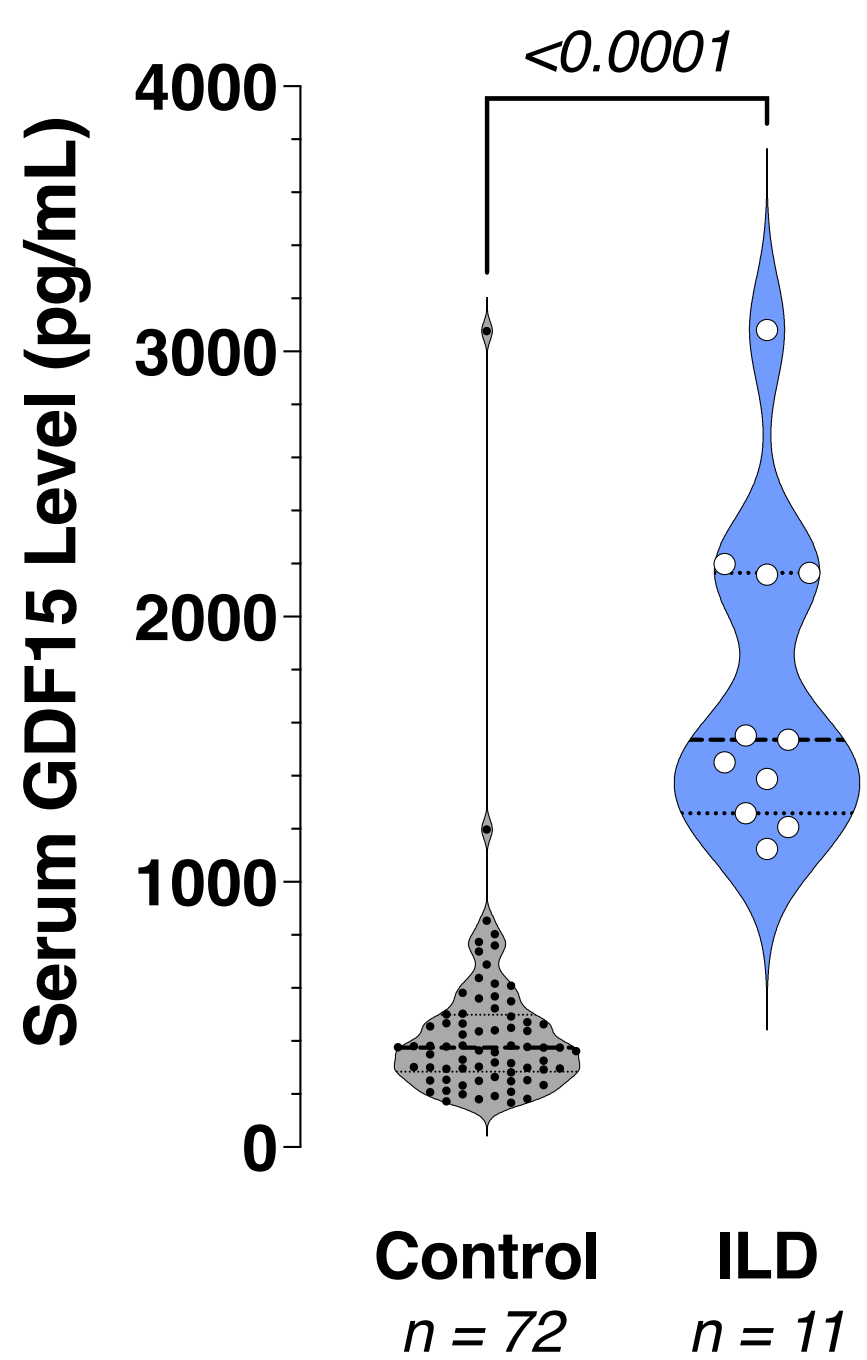

Supplemental Figure 2B. GDF15 levels of ILD patients whose histology was examined in Figure 2. Statistical analysis: Kolmogorov-Smirnov test.

C

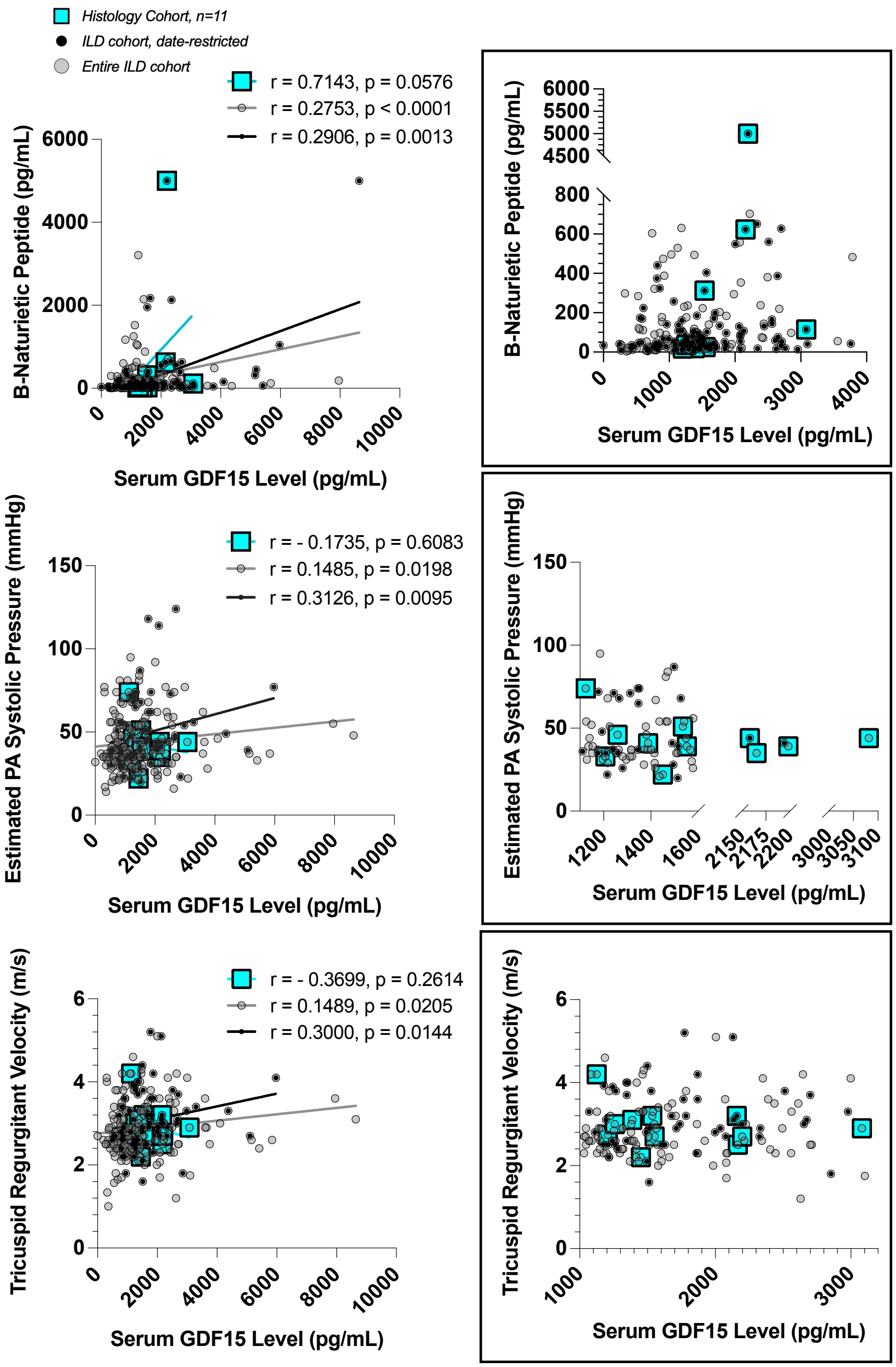

Supplemental Figure 2C. BNP, ePASP, and TRV values of ILD patients whose histology was examined in Figure 2, highlighted within the entire ILD cohort. "Date-restricted" refers to the portion of the cohort whose values of interest were obtained within 24 months of the GDF15 level. BNP: total n=200, date-restricted=119; ePASP: total n=246, date-restricted=68; TRV: total n=242, date-restricted=66. Statistical analyses: Spearman correlation.

D

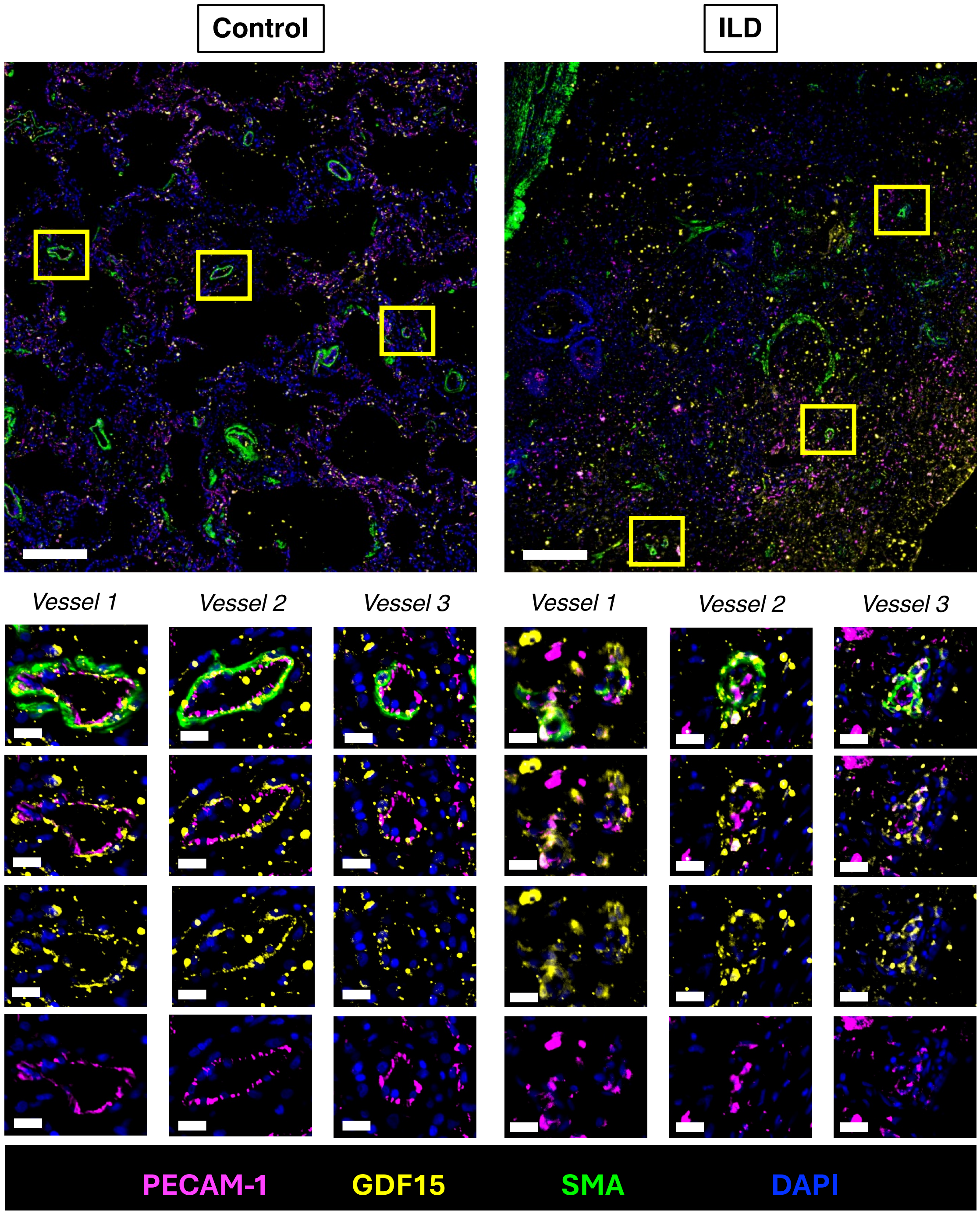

Supplemental Figure 2D. Representative immunofluorescence images from control and ILD patients, stained for SMA (green), PECAM-1 (pink), GDF15 (yellow), and counterstained with DAPI (blue). Partial zoom scale bar = 250 um; full zoom scale bar = 20 um.

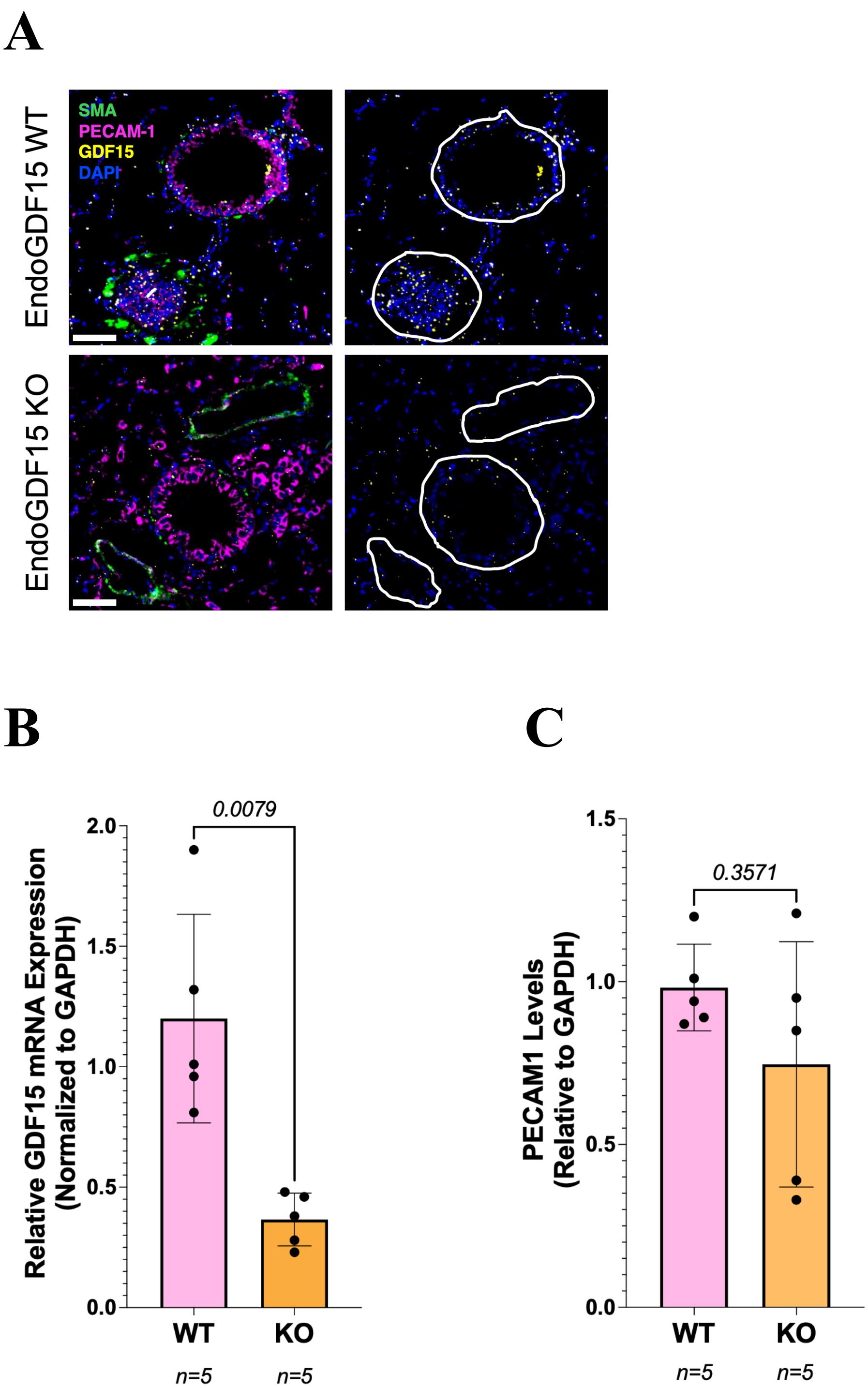

Supplemental Figure 3. (A) Representative RNA scope images of murine histologic specimens from saline-treated mice. N=5 per group. Scale bar = 50 um. **(B)** Quantification of lung endothelial *Gdf15* mRNA via qPCR following murine lung tissue digestion and endothelial cell isolation, normalized to *Gapdh* (n=5 per group). **(C)** Quantification of lung endothelial *Pecam-1* mRNA levels normalized to *Gapdh* (n=5 per group). Statistical analysis: Kolmogorov-Smirnov test (B, C).

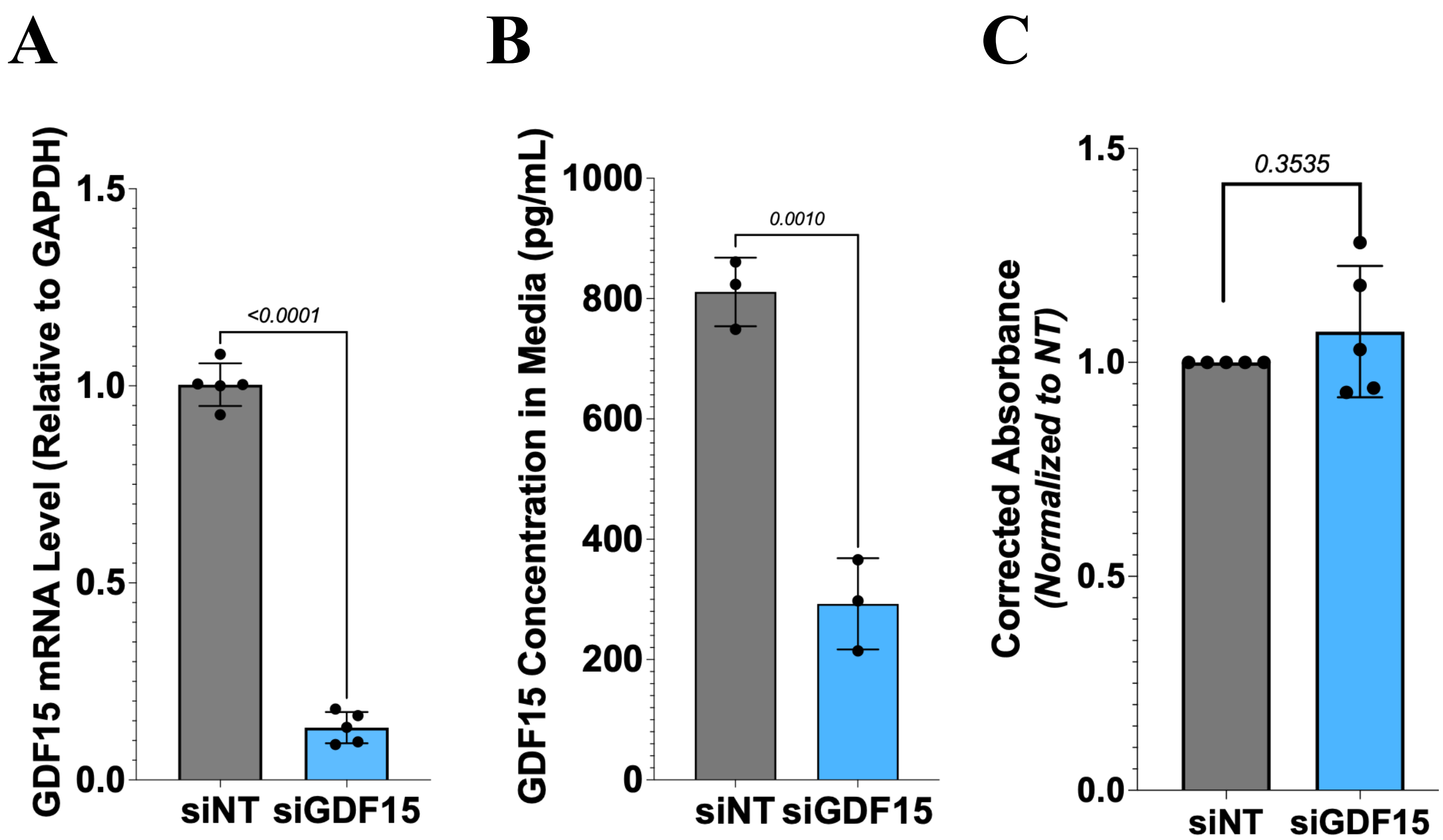

Supplemental Figure 4. (A) *GDF15* mRNA levels measured by qPCR in siRNA-HPMVECs over five separate representative *GDF15* knockdown experiments. **(B)** Quantification of GDF15 in the media of confluent siRNA-HPMVECs over 48H. **(C)** Measurement of cell viability with WST Assay of siRNA-HPMVECs. Statistical analysis: Welch's t-test (A, B, C).

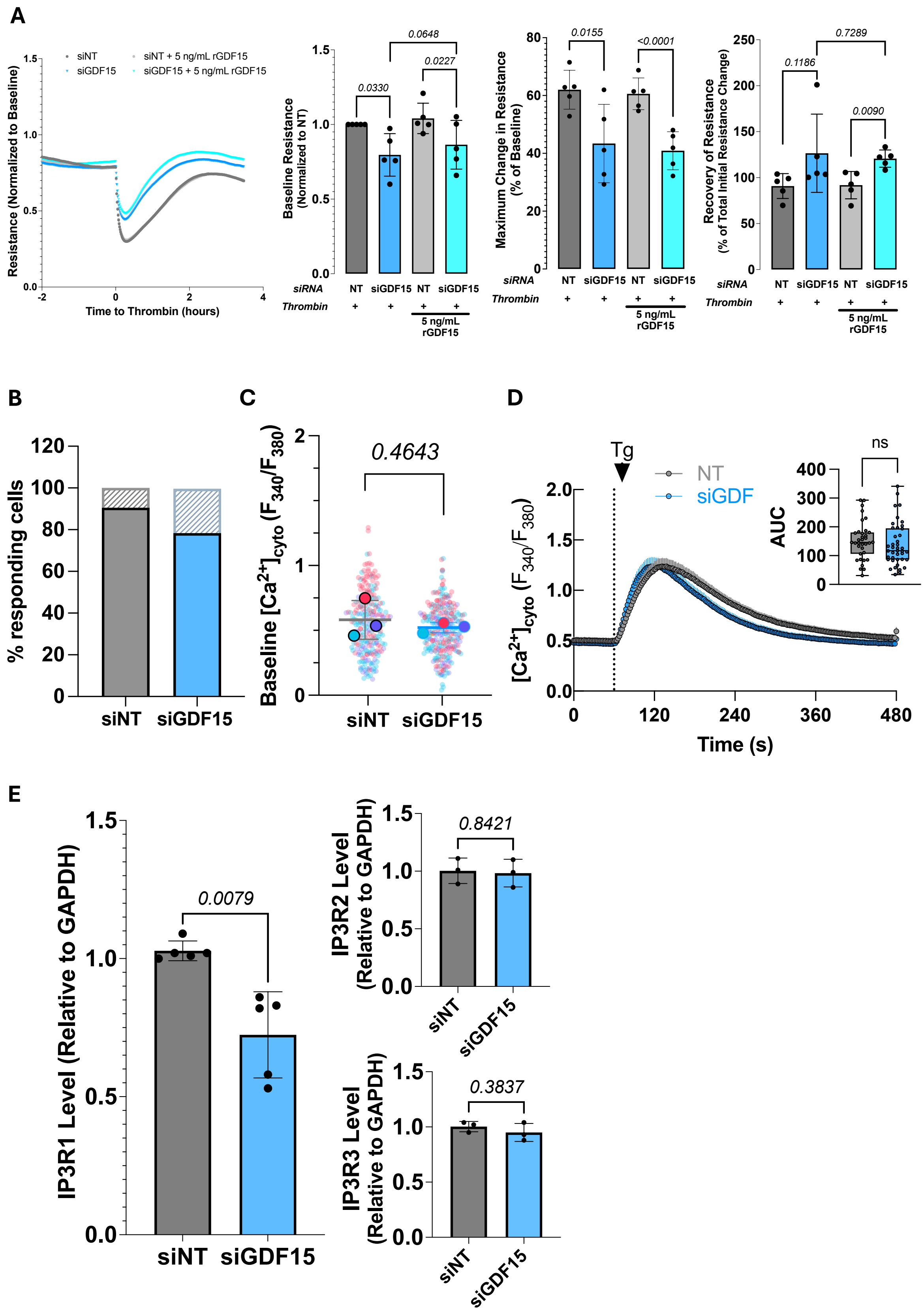

A
siNT
siGDF15
siNT + 5 ng/mL rGDF15
siGDF15 + 5 ng/mL rGDF15
Resistance (Normalized to Baseline)
Time to Thrombin (hours)
Baseline Resistance (Normalized to NT)
0.0648
0.0330
0.0227
Maximum Change in Resistance (% of Baseline)
0.0155
<0.0001
Recovery of Resistance (% of Total Initial Resistance Change)
0.7289
0.1186
0.0090
siRNA
Thrombin
NT
siGDF15
5 ng/mL rGDF15
B
% responding cells
C
Baseline [Ca2+]cyto (F340/F380)
0.4643
D
Tg
NT
siGDF
ns
AUC
[Ca2+]cyto (F340/F380)
Time (s)
E
IP3R1 Level (Relative to GAPDH)
0.0079
IP3R2 Level (Relative to GAPDH)
0.8421
IP3R3 Level (Relative to GAPDH)
0.3837
siNT
siGDF15

Supplemental Figure 5. (A) *GDF15* knockdown in HPMVECs is associated with decreased change in permeability in response to insult, the effects of which are not altered with pre-treatment of cells with rGDF15. **(B)** Percentage of cells responding to thrombin treatment during epifluorescence Ca^{2+} imaging. **(C)** Quantification of baseline cytosolic Ca^{2+} levels measured over 60 seconds prior to thrombin treatment. **(D)** ER Ca^{2+} content: Representative traces of cytosolic Ca^{2+} levels in HPMVECs over time, showing responses to thapsigargin treatment. *Inset:* area under the curve, calculated as the ratio of Fura-2 fluorescence intensity at 340 nm to intensity at 380 nm excitation. Data were pooled from two independent experiments. **(E)** *IP3R1, IP3R2,* and *IP3R3* mRNA levels measured by qPCR in siRNA-HPMVECs over five separate representative *GDF15* knockdown experiments (*IP3R1)* or one knockdown experiment (*IP3R2, IP3R3*). Statistical analysis: paired t-test (A), Mann-Whitney U test (C, D), Kolmogorov-Smirnov test (E).

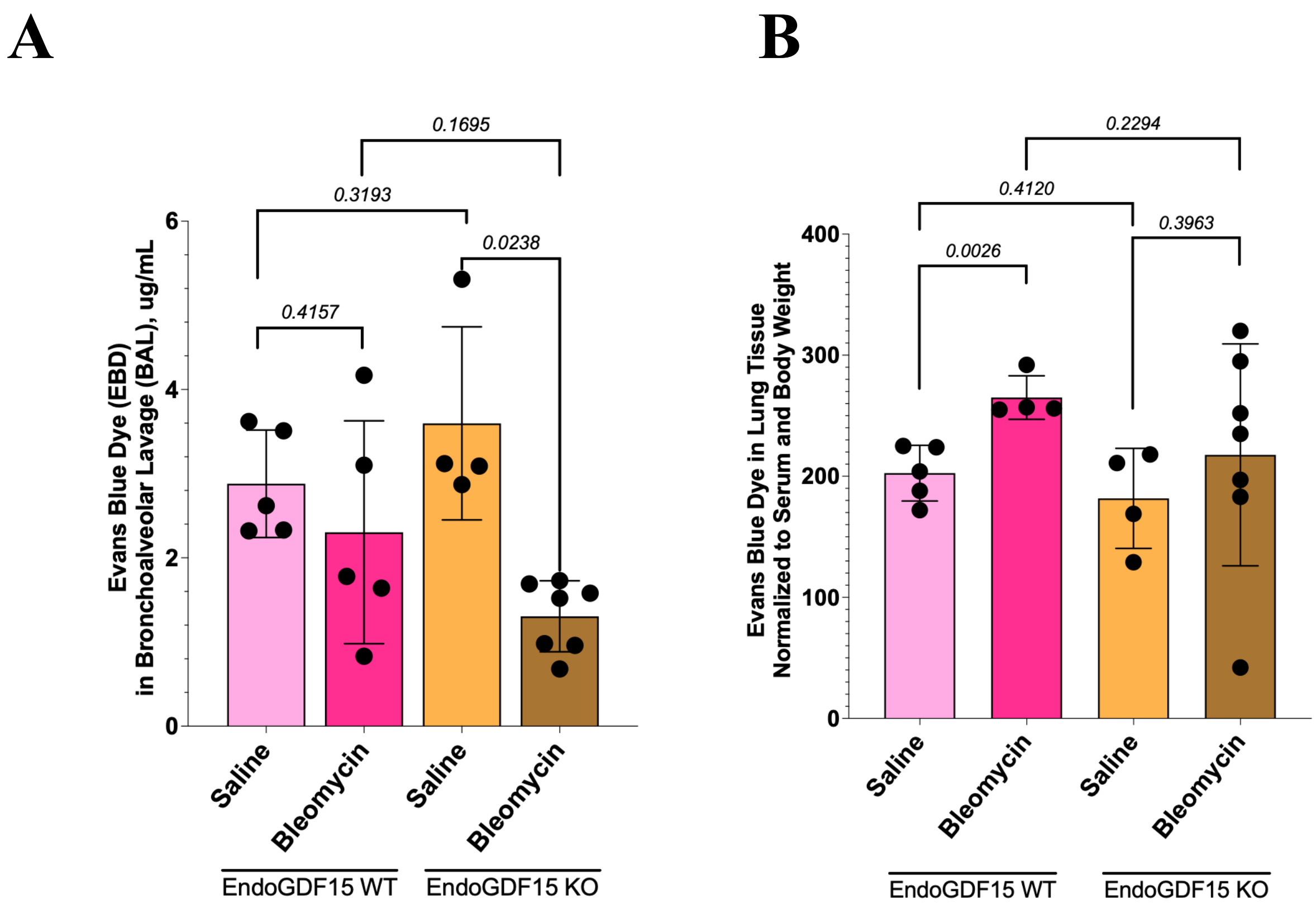

Supplemental Figure 6. Murine endothelial *Gdf15* knockout blunts dye accumulation in the pulmonary parenchyma following injury with intratracheal bleomycin. **(A)** Quantification of dye in bronchoalveolar lavage fluid following sacrifice at day 7. **(B)** Quantification of dye in lung tissue, normalized to serum dye levels and body weight. Statistical analysis: Welch's t-test (A, B).
